# Burden of Hypertensive Heart Disease and Its Risk Factors in East Asia, 1990–2021: Findings From the Global Burden of Disease Study 2021

**DOI:** 10.5334/gh.1472

**Published:** 2025-09-22

**Authors:** Zhongqing Zhou, Zixiang Ji, Jiazhe Hou, Jing Yang, Hengjing Wu, Lijuan Zhang

**Affiliations:** 1 Clinical Center for Intelligent Rehabilitation Research, Shanghai YangZhi Rehabilitation Hospital (Shanghai Sunshine Rehabilitation Center), Tongji University School of Medicine, Tongji University, Shanghai 201613, China; 2 School of Public Health and General Practice Medicine, Tongji University School of Medicine, Tongji University, Shanghai 200331, China

**Keywords:** East Asia, hypertensive heart disease, prevalence, mortality, disability-adjusted life-years, trends, risk factors

## Abstract

**Introduction:**

Long-term hypertensive heart disease (HHD) trends in East Asia offer insights for heart disease prevention. We analyzed HHD burden trends in East Asia (1990–2021).

**Methods:**

We analyzed trends in age-standardized prevalence (ASPR), death, and disability-adjusted life-years (DALYs) rates of HHD in East Asia from 1990 to 2021 using data from the Global Burden of Disease Study 2021. Annual average percent changes (AAPC) were calculated via Joinpoint regression. Relative risks were estimated via population attributable fraction (PAF).

**Results:**

In 2021, East Asia reported 4,354,899 prevalent cases of HHD, 361,483 deaths and 6,079,780 DALYs. East Asia has seen a decrease in the overall prevalence of hypertensive heart disease (HHD) from 1990 to 2021, yet the ASPR for HHD has increased in the last decade, from 2012 to 2021. During this period, developed areas such as Japan have observed a growing trend of HHD among younger demographics. In contrast, developing regions like North Korea continue to face challenges in managing the condition effectively. The burden of HHD is particularly pronounced among females over 75 years of age, while males exhibit higher risk due to poor lifestyle factors. From 1990 to 2021, the PAF due to high body mass index (BMI) consistently increased across East Asia, with the following AAPC and 95% confidence interval (95% CI): China (1.55, 95%CI: 1.54, 1.56), Japan (0.79, 95%CI: 0.78–0.81), South Korea (0.86, 95%CI: 0.85, 0.86), China-Taiwan (1.3, 95%CI: 1.28, 1.33), North Korea (1.55, 95%CI:1.54, 1.55), and Mongolia (0.24, 95%CI: 0.23, 0.25).

**Conclusions:**

East Asia faces a significant HHD prevalence, with elderly females needing particular focus. High BMI is a notable risk factor. Given the differing HHD impacts across regions, targeted strategies that consider regional and national differences are essential for reducing the burden.

## 1. Introduction

Hypertension is the primary cause of cardiovascular diseases (CVD) and represents a major global public health burden (1). Approximately 1.4 billion adults worldwide have hypertension, projected to exceed 1.6 billion by 2025 (2). Hypertensive heart disease (HHD), characterized by cardiomyocyte hypertrophy and interstitial fibrosis, occurs as the heart adapts to sustained pressure elevation, leading to left ventricular hypertrophy and dilatation (3,4). As a major complication of hypertension, HHD often progresses to severe cardiovascular events, including heart failure, myocardial infarction, and cardiac arrhythmias (5), profoundly impacting patientsʼ quality of life. In 2019, HHD accounted for 19.6 million cases, 1.16 million deaths and 21.5 million disability-adjusted life-years (DALYs) globally (6).

The epidemiology of HHD varies across geographic and demographic contexts (7). Notably, East Asia bore a heavy HHD burden in 2019, with the worldʼs highest age-standardized prevalence of 426 cases per 100,000 population (8). Projections show East Asiaʼ HHD-related deaths will rank the third among CVD by 2050, with an estimated 7 deaths per 100,000 population (9). Aging populations amplify CVD burden (10), and East Asiaʼs rapid demographic aging has fueled rising HHD prevalence (11,12). Additionally, high body mass index (BMI) and excessive dietary salt intake further elevate hypertension and heart disease risks (2). East Asia is a dynamic and diverse region, home to about 20% of the global population. It includes developed nations like Japan and South Korea, as well as developing countries such as China, North Korea, and Mongolia. These countries share notable cultural and ethnic similarities. Given these factors, the impact of HHD on CVD health issues in East Asia is significant. Targeted regional studies are crucial for effective prevention and management strategies.

While numerous global studies have assessed the burden of HHD (7,13), few have focused specifically on the entire East Asian region (14,15). Previous HHD risk factors have mainly measured burden within specific cross-sections, not how these risk factors change over time (8,16). Our data are from the Global Burden of Disease Study 2021 (GBD 2021). We use them to analyze temporal trends in East Asian HHD burden (1990–2021) and explore risk factor dynamics. This study seeks to understand HHD’s East Asian impact and provide policymakers with insights to optimize healthcare resources and reduce regional chronic disease burdens.

## 2. Methods

### 2.1 Data source

GBD 2021 comprehensively analyzes disease and injury burdens in 204 countries/territories. It disaggregated health outcomes by sex/age for 371 conditions and 88 risk factors, enabling granular insights into global health trends. Key metrics (incidence, prevalence, mortality, DALYs) offer a holistic view of disease burden (17). GBD 2021 uses diverse data sources, including census data, vital statistics, disease registries, satellite sensing, and air quality monitoring. To ensure reliability, this diverse data integration is supplemented by systematic searches of government data, international databases, and peer-reviewed literature (18). Advanced analytical techniques help ensure data accuracy. Spatiotemporal Gaussian process regression is used to interpolate missing data, and the Bayesian meta-regression tool called DisMod-MR is applied to model global disease epidemiology (19).

### 2.2 Indicators utilized

#### (1) Key metrics

HHD is identified using codes from the 9th and 10th revisions of the International Classification of Diseases and Injuries (ICD-9: 402–402.91; ICD-10: I11–I11.2, I11.9) (19). This study examines key metrics including prevalence, death, and DALYs, each accompanied by 95% uncertainty interval (95% UI) to indicate the confidence in the estimates. For temporal trends, we analyzed trends in age-standardized prevalence (ASPR), death (ASDR), and disability-adjusted life-years (ASDALR) rates of HHD. Data for analysis are sourced from the Global Burden of Disease Study website (https://ghdx.healthdata.org/gbd-results-tool).

#### (2) Average annual percentage change (AAPC)

We used AAPC and its 95% confidence interval (95% CI) to assess dynamic changes. AAPC is calculated via Joinpoint regression. This method identifies breakpoints in time series data to define consecutive segments, calculates the annual percent change (APC) for each segment, and averages APC values weighted by segment duration to quantify overall trend changes (20).

#### (3) Risk factors

Our study investigates the risk factors associated with DALYs in the context of GBD 2021 framework. The GBD 2021 classifies risk factors for HHD into a three-level structure. Level 1 includes metabolic risks, dietary risks, behavioral risks, and environmental/occupational risks; Level 2 includes high systolic blood pressure (SBP), high BMI, diet low in fruits and vegetables, diet high in sodium, high alcohol use, non-optimal temperature, other environmental risks; and Level 3 includes high temperatures, low temperatures, and lead exposure.

#### (4) Population attributable fraction (PAF)

PAF measures the proportion of disease or mortality in a population associated with a specific risk factor. It indicates the theoretical reduction in disease burden if the risk factor were removed (21). GBD studies compute PAF using integrated modeling. This method uses data-driven approaches to identify risk-disease relationships and theoretical minimum risk exposure levels. It quantifies relative risk (RR) from cohort studies. The method combines RR with population exposure data to estimate avoidable disease burden from eliminating risks (22).

#### (5) Sociodemographic Index (SDI)

SDI measures socioeconomic development’s health impact using geometric mean of three indicators: total fertility rate under 25, mean education years for ≥15 population, and lag-distributed income per capita. In GBD 2021, SDI scores has been adjusted to 0–100, where higher values indicate better socioeconomic status (23). SDI data are accessible via: https://ghdx.healthdata.org/record/global-burden-disease-study-2021-gbd-2021-socio-demographic-index-sdi-1950–2021.

### 2.3 Statistical analysis

We assessed trends in ASPR/ASDR/ASDALR, PAF for risk-attributable DALYs, and ASDALR using AAPC. HHD burden was analyzed by age (<5 to 95+ years) and gender (female/male/both). Age groups were consolidated into four categories (15–44, 45–59, 60–74, >75 years) for compositional analysis. The 1990–2021 period was divided into three phases (1990–2000, 2001–2011, 2012–2021) to examine East Asian HHD trends. Analyses used R 4.4.2 for data processing and visualization, with Joinpoint 5.0 for regression. SDI-ASR correlations were tested using Pearson’s method. Statistical significance threshold was set at two-sided *P* < 0.05.

## 3.Results

### 3.1 The burden and trends of HHD in East Asia from 1990 to 2021

In 2021, East Asia recorded 4,354,899 cases of HHD, 361,483 HHD-related deaths, and 6,079,780 HHD-associated DALYs cases. The ASPR of HHD ranged from 51.94 per 100,000 population in Mongolia (95% UI: 34.09, 75.66) to 230.62 per 100,000 in China-Taiwan (95% UI: 178.4, 301.01). The ASDR of HHD varied from 2.56 per 100,000 in Japan (95% UI: 2.02, 2.88) to 26.83 per 100,000 in North Korea (95% UI: 19.35, 36.15). Similarly, the ASDALR due to HHD ranged from 40.95 per 100,000 in Japan (95% UI: 35.52, 44.6) to 465.32 per 100,000 in North Korea (95% UI: 336.69, 611.31) (Table 1).

**Table 1:** Number and ASR of prevalence, mortality, and DALYs of HHD per 100,000 people in East Asia between 1990 and 2021.


	1990		2021		1990–2021
		
	NUMBER (95% UI)	ASR (95% UI)	NUMBER (95% UI)	ASR (95% UI)	AAPC(95%CI)

**Prevalence**					

China	1,503,019 (1,161,178–1,916,382)	218.24 (169.31,274.83)	3,912,158 (2,989,417–5,056,002)	192.47 (146.68,245.02)	–0.44 (–0.46,–0.42)

Japan	75,062 (51,652–100,265)	48.72 (34.43,64.72)	195,613 (140,989–264,657)	44.91 (33.64,58.26)	–0.41 (–0.49,–0.32)

South Korea	28,327 (21,349–36,057)	107.71 (78.22,142.23)	91,856 (71,867–116,578)	103.01 (81.11,129.99)	–0.19 (–0.23,–0.15)

China-Taiwan	26,792 (20,270–35,311)	196.57 (147.5,260.35)	99,331 (76,001–129,684)	230.62 (178.4,301.01)	0.51 (0.46,0.56)

North Korea	20,489 (15,857–26,581)	160.25 (121.53,211)	54,979 (42,119–71,899)	181.41 (139.98,237.86)	0.39 (0.39,0.40)

Mongolia	522 (354–728)	53.59 (35.77,75.28)	962 (662–1362)	51.94 (34.09,75.66)	–0.10 (–0.11,–0.08)

**Death**					

China	232,479 (155,806–275,584)	42.64 (30.32,49.56)	328,119 (224,717–425,288)	18.85 (12.89,24.47)	–0.80 (–0.83,–0.77)

Japan	13,658 (11,918–14,608)	9.58 (8.21,10.32)	15,943 (11,809–18,385)	2.56 (2.02,2.88)	–2.61 (–2.70,–2.55)

South Korea	3,215 (2,109–3,826)	18.08 (12.81,21.49)	4,888 (3,500–7,596)	5.63 (4,8.66)	–4.10 (–4.36,–3.87)

China-Taiwan	2,685 (2,537–2,813)	23.78 (21.72,25.18)	4,921 (4,180–5,406)	10.91 (9.36,11.99)	–3.77 (–3.92,–3.65)

North Korea	3,247 (2,033–4,546)	29.48 (18.79,43.56)	7,460 (5,358–10,092)	26.83 (19.35,36.15)	–0.31 (–0.32,–0.29)

Mongolia	126 (79–185)	13.8 (8.63,20.25)	152 (102–216)	9.01 (6,12.88)	–1.40 (–1.51,–1.25)

**DALYs**					

China	4,971,332 (3,273,139–5,916,486)	716.02 (488.69,841.36)	5,589,287 (3,973,271–7,160,215)	292.54 (208.19,374.2)	–0.95 (–0.97,–0.92)

Japan	210,802 (190,712–222,341)	137.7 (123.13,145.91)	193,598 (156,853–218,427)	40.95 (35.52,44.6)	–2.90 (–3.00,–2.83)

South Korea	68,498 (42,624–79,448)	284.17 (192.22,332.66)	66,592 (51,734–107,310)	75.36 (58.54,119.53)	–3.89 (–4.12,–3.71)

China-Taiwan	55,225 (52,722–57,861)	404.91 (381.23,424.23)	84,569 (75,170–91,762)	198.02 (177.78,214.35)	–4.27 (–4.35,–4.19)

North Korea	68,064 (40,911–97,438)	504.5 (315.76,705.23)	142,229 (101,840–187,748)	465.32 (336.69,611.31)	–0.26 (–0.27,–0.25)

Mongolia	2,744 (1,715–4,033)	273.93 (170.81,401.72)	3,505 (2,406–4,943)	167.89 (114.29,236.21)	–1.58 (–1.74,–1.45)


AAPC average annual percent change, ASR age-standardized rates, CI confidence interval, DALYs disability-adjusted life years, UI uncertainty interval.

**Figure 1:**
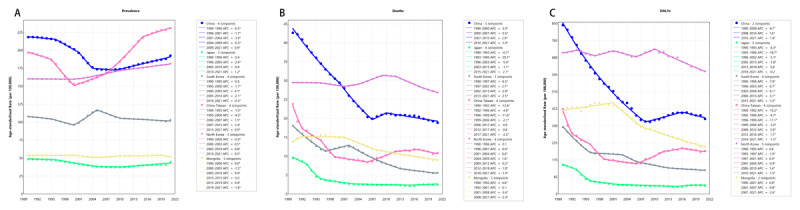
Temporal trends in ASR of HHD in East Asia from 1990 to 2021. **(A)** Age standardized prevalence rate. **(B)** Age standardized death rate. **(C)** Age standardized DALYs rate. HHD, hypertensive heart disease. DALYs, disability-adjusted life-years.

From 1990 to 2021, regional variations in the burden of HHD remained minimal. The ASPR increased only in China-Taiwan (AAPC: 0.51, 95% CI: 0.46, 0.56) and North Korea (AAPC: 0.39, 95% CI: 0.39, 0.40), whereas ASPR, ASDR, and ASDALR declined across all other regions. However, regional differences widened over the past 10 years (2012–2021). ASPR declined only in South Korea and Mongolia. It increased in China, Japan, Taiwan, and North Korea. In Japan, ASDR and ASDALR also reversed to upward trends (Figure 1, Table S1, Figure S1–S2).

We further analyzed trends of HHD across East Asian by subgroup from 2012 to 2021. In the gender-stratified analysis, the burden of HHD was more pronounced among males than females in Japan, South Korea, and China-Taiwan. Notable AAPC differences in Japan: ASPR 1.71(male) vs 1.01(female), ASDR 1.91 vs –0.61, ASDALR 2.16 vs 0.52 (Table S2–S3). In the age-specific analysis, HHD prevalence in China-Taiwan increased across all age groups. In Japan, HHD-related mortality and DALYs rose among individuals aged 35–80 years. Mongolia witnessed upticks in HHD prevalence, mortality, and DALYs in 85–89 age group. Additionally, China-Taiwan’s 15–19 age group was the first to demonstrate elevated prevalence and DALYs in 2021 compared with 2012 (Figure S3–S5).

**Figure 2:**
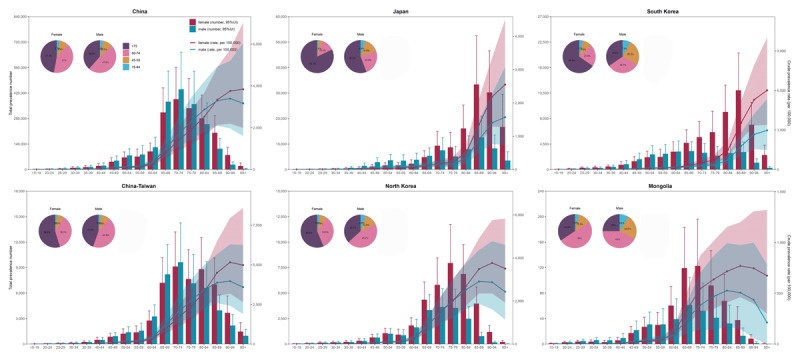
East Asian HHD prevalence cases in 2021, by age and sex. The crude prevalence rates and their 95% uncertainty intervals are shown in the line graphs. The male and female prevalence composition by age group are displayed in pie charts.

Older adults carried a heavier HHD burden than younger generations. Those individuals aged 75 years and older accounted for 44.7% of all HHD cases, 50.9% of HHD-related DALYs, and 70.6% of HHD deaths (Figure S6). Within the 75+ age group, females bore a heavier burden, whereas in younger people under 60 years old, males bore a greater burden. Japan exhibited the highest HHD prevalence among adults aged 75 years and older, with rates reaching 82.1% in females and 55.8% in males. In contrast, Mongolia had the lowest in this age group, at 34.2% in females and 25% in males (Figure 2, Figure S7–S8).

### 3.2 Analysis of attributable risk factors

**Figure 3:**
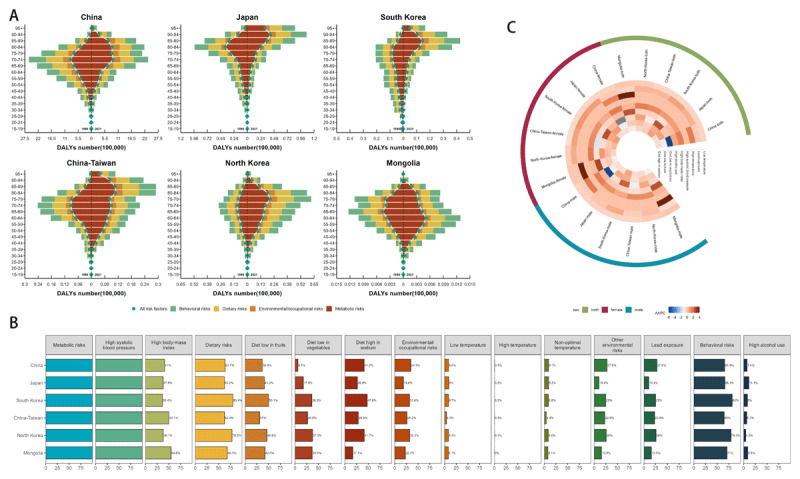
**(A)** DALYs for risk factors at level 1 for each age group. **(B)** The PAF of DALYs for HHD in East Asia in 2021. **(C)** AAPC in PAF of DALYs for Non-level 1 Risk Factors for HHD in East Asia from 1990 to 2021. PAF, population attributable fraction. AAPC, average annual percentage changes.

In East Asia, the age groups with the highest HHD-related DALYs showed regional differences between 2021 and 1990. The trend remained unchanged in China. It shifted to younger populations in Mongolia. All other regions saw a shift to older age groups. Metabolic factors were the leading risk for HHD across East Asia from 1990 to 2021 (Figure 3A). In South Korea and Japan, the DALYs attributed to HHD were significantly lower among males compared to females (Figure S9-S10).

In 2021, the PAF for level 1 risk factors varied across East Asia (excluding metabolic factors). China had the highest PAF for environmental/occupational risks (34.3%), while South Korea had the highest PAF for dietary (80.4%) and behavioral (82.0%) risks. The PAF for level 2 and level 3 risk factors also varied across the region (excluding high systolic blood pressure). high sodium diet was a significant risk factor in China, low fruit intake was prominent in South Korea, Japan, and North Korea, and high BMI was most notable in China-Taiwan and Mongolia (Figure 3B).

Gender differences do exist. Males generally exhibited higher PAF values of behavioral, dietary, and environmental/occupational risks compared to females in all regions except Japan. Furthermore, males were more frequently exposed to high alcohol intake, lead exposure, high sodium diet, low fruit intake, and low vegetable intake. Conversely, females were more likely to have a high BMI (Figure S11).

From 1990 to 2021, the trends in the PAF for risk factors across East Asia showed notable variations. The fastest-rising risk factors in each region were as follows: high BMI in Japan (AAPC: 0.79, 95% CI: 0.78, 0.81), China-Taiwan (AAPC: 1.3, 95% CI: 1.28, 1.33), and North Korea (AAPC: 1.55, 95% CI: 1.54, 1.55); high temperature in China (AAPC: 1.56, 95% CI: 0.62, 2.71); low vegetable intake in South Korea (AAPC: 2.46, 95% CI: 2.41, 2.51); and high alcohol intake in Mongolia (AAPC: 2.45, 95% CI: 2.36, 2.56). Notably, high BMI continued to rise across all East Asian regions (Figure 3C, Figure S12).

In terms of ASDALR for HHD attributable to risk factors, high BMI in North Korea showed an increase (AAPC: 1.28, 95% CI: 1.27, 1.29). This increase was observed in both females and males. Additionally, lead exposure (AAPC: 0.04, 95% CI: 0.03, 0.05) also increased in North Korea, primarily driven by an increase in female (AAPC: 0.32, 95% CI, 0.30, 0.34). In Mongolia, high alcohol consumption (AAPC: 0.76, 95% CI: 0.68, 0.84) has increased, and this trend was also observed in both females and males (Table S4–S6).

### 3.3 Association Between SDI and Disease Burden

East Asia (1990–2021) showed a strong negative SDI-ASDALR correlation (R = –0.86, *P* < 0.001), with rising SDI linked to ASDALR decline. SDI also negatively correlated with ASDR (R = –0.8, *P* < 0.001) and ASPR (R = –0.48, *P* = 0.002), showing both metrics declined as SDI rose (Figure S13).

## 4. Discussion

This study looked at how HHD trends have changed in East Asia from 1990 to 2021. The results revealed a mixed picture. Overall, the total burden of HHD in the region decreased during this period. But in recent years, the ASPR has gone back up. North Korea bears a more serious HHD problem. In developed places like Japan and China-Taiwan, HHD is starting to affect people at an earlier age. Older females are hit especially hard by HHD. Females face more metabolic risks, while males have higher behavioral risks. From 1990 to 2021, high BMI has been rising steadily.

From 2012 to 2021, the ASPR of HHD in East Asia increased, which might be attributed to the following reasons. First, rapid economic growth has driven substantial lifestyle changes, including increased consumption of high-calorie, high-fat diets and reduced physical activity, leading to rising rates of obesity, hyperlipidemia, and hypertension (24). China, with its large population, is especially affected (25). Second, the aging population in East Asia has heightened the risk of HHD, as older individuals are more susceptible to the disease, resulting in a greater number of cases (26). Third, advancements in diagnostic technology have led to more precise and prompt HHD identification (27). Things like telemedicine and wearable devices have also improved how we monitor and diagnose high blood pressure and related heart problems (28). Finally, the American Heart Association’s 2017 decision to lower the blood pressure threshold from 140/90 mmHg to 130/80 mmHg has contributed to the observed rise in recorded HHD cases (29). These factors collectively highlight the urgent need for enhanced prevention strategies and improved disease management to address the growing burden of HHD in the region.

The impact of HHD varies significantly between Japan and North Korea due to differences in their healthcare systems and social development. Japan, with its high SDI and advanced medical resources, is well-equipped to manage hypertension effectively (30). In contrast, North Korea faces challenges such as limited social development and scarce medical resources, which hinder effective blood pressure control and increase the risk of HHD (10). For developing nations like North Korea, the challenges extend beyond inadequate treatment capacity. The lack of screening and diagnostic tools can lead to underestimation of hypertension prevalence, potentially hiding a more severe reality (31). This disparity underscores the importance of strengthening primary healthcare systems and ensuring equitable resource distribution. Tailoring policies to address local contexts could play a crucial role in reducing the burden of HHD in East Asia.

Managing high blood pressure is a challenge for many developing countries. In Africa, for instance, approximately 30% of the population suffers from hypertension. However, only 27% of these individuals are aware of their condition, and a mere 18% receive treatment. Alarmingly, just 7% successfully control their blood pressure (32). Despite limited healthcare resources, there are opportunities for improvement. A research project in China demonstrated that simple, low-cost initiatives led by non-doctors in rural areas can effectively manage high blood pressure and reduce the risk of CVD (33). Given the proximity and potential similarities in healthcare challenges between China and North Korea, such an approach could offer valuable lessons for improving hypertension management in North Korea.

There are significant gender differences in the burden of HHD. Older females are more likely to experience the burden of HHD compared to older males, while younger males are more affected by the condition than younger females. Research indicates that males often engage in unhealthier behaviors, such as excessive alcohol consumption, which significantly increases their risk of CVD (34). Females, on the other hand, naturally produce estrogen, which provides protection for their cardiovascular system. This explains why males tend to develop HHD at a younger age. However, as females grow older and go through menopause, the hormonal changes in their bodies increase their risk of HHD (35,36). These differences highlight the need for targeted approaches to address HHD in each group. For males, prioritizing lifestyle changes is crucial: quitting smoking, reducing alcohol intake, and managing blood pressure. For females, it is important to monitor bodily changes closely as they age.

In East Asia, the prevalence and mortality rates of HHD increase significantly with age in both males and females. The region is also experiencing rapid population aging: by 2030, the proportion of people aged 60 and over is projected to increase from 12.9% in 1990 to 18.1% (37). As the population grows older, effectively preventing and managing HHD in older adults becomes even more critical.

Notably, in more developed parts of East Asia, such as Japan and China’s Taiwan region, the burden of HHD has shifted toward younger populations over the past decade (2012–2021). Studies reveal that while deaths linked to high blood pressure have decreased for middle-aged and older adults, the improvement has been less pronounced among younger individuals (38). This shows that even in areas with better medical resources, managing long-term diseases is still a challenge. Also, HHD is becoming more common among younger people, which is something that was usually seen in older people.

Hypertension is the primary cause of HHD, as confirmed by this study. In fact, it accounts for all the risk associated with losing healthy life years due to HHD. Excessive salt consumption contributes to 29.1% of the global risk for HHD (8,16). In East Asia, particularly in countries such as China, South Korea, and North Korea, the risk is significantly higher. This is likely due to the high consumption of salty foods like kimchi and salty snacks in these regions (39).

High BMI plays a crucial role in health outcomes. The global rise in obesity is primarily driven by increasing obesity rates in Asia, particularly in China and Japan (40). This trend is largely attributed to changes in dietary patterns, which now include diets high in fat (especially saturated fat), cholesterol, refined carbohydrates, and low in fiber. Additionally, sedentary lifestyles and high stress levels further contribute to the growing prevalence of obesity in the region (25). In the future, managing obesity may become as critical as managing hypertension in controlling HHD.

To reduce these risk factors, several strategies can be implemented. First, it is crucial to educate communities about the risks associated with hypertension and high BMI. Promoting diets that are low in salt and high in fiber, along with encouraging regular physical activity, are essential steps. Reducing salt intake is particularly important, as it has a significant impact on blood pressure levels and the incidence of HHD (41). The World Health Organization recommends limiting daily salt intake to 5 grams, highlighting its importance in maintaining cardiovascular health (42). Notably, adopting dietary patterns similar to the traditional Japanese diet, which is rich in soy products, fish, seaweed, fruits, vegetables, and green tea, has been associated with a lower risk of CVD mortality (43). This dietary approach not only emphasizes nutrient-rich foods but also aligns with guidelines that promote overall well-being. When it comes to policymaking, it is important for governments to consider the overall population when developing guidelines. By assessing the prevalence and impact of these risk factors, policymakers can create targeted public health strategies that address specific needs. This tailored approach ensures that policies are effective and relevant to the communities they serve (44,45).

This study has several advantages. First, it utilizes high-quality data from the GBD database. These data encompass a wide geographical area and population, providing a robust foundation for the analysis. Second, it represents the first investigation into the burden of HHD in East Asia. The study provides detailed insights into the prevalence, mortality, DALYs, and risk factors of HHD across six countries/regions in East Asia. Furthermore, it reveals significant findings regarding the burden of HHD in the region.

There are also limitations. First, the GBD data relies on model estimates in regions with limited resources, which may lead to inaccuracies. Second, differences in data collection methods, reporting practices, disease definitions, and diagnostic criteria across regions can affect the consistency and accuracy of the estimates. While the GBD data provides valuable insights, the results should be interpreted with caution. Future research should supplement these estimates with direct monitoring data whenever possible to enhance the accuracy of global disease burden assessments. To address these challenges, we took several steps to maximize accuracy. The data were rigorously cleaned during analysis, and subgroup analyses were conducted by age group, gender, and year. Additionally, risk factors were dynamically assessed throughout the study period.

## 5. Conclusion

East Asia faces a considerable HHD burden. While the ASDR and ASDALR for HHD dropped from 1990 to 2021, ASPR rose between 2012 and 2021. Females over 75 are crucial for intervention efforts. Besides hypertension, high BMI is a key HHD risk factor. Policies should be customized to fit local needs. To tackle the increasing HHD burden, interventions like early screening, targeting specific groups, managing hypertension and BMI, and health education are recommended.

## Data Accessibility Statement

The datasets used in this study are publicly accessible and could be viewed using the GBD Results Tool at https://ghdx.healthdata.org/gbd-results-tool.

## Additional File

The additional file for this article can be found as follows:

10.5334/gh.1472.s1 Supplementary Files. Table s1 to s6 and Figures s1 to s13.
