# Supplementary material for: Burden of Hypertensive Heart Disease and Its Risk Factors in East Asia, 1990–2021: Findings From the Global Burden of Disease Study 2021
Source: Glob Heart. 2025 Sep 22;20(1):82. doi: 10.5334/gh.1472 (PMC12466111; doi:10.5334/gh.1472)

Table S1

AAPC of ASPR, ASDR and ASDALR for HHD in East Asia from 1990 to 2021

	China	Japan	South Korea	China-Taiwan	North Korea	Mongolia
ASPR(95%CI)						
1990-2021	-0.44	-0.41	-0.19	0.51	0.39	-0.1
	(-0.46,-0.42)	(-0.49,-0.32)	(-0.23,-0.15)	(0.46,0.56)	(0.39,0.4)	(-0.11,-0.08)
1990-2000	-0.86	-1.22	-1.17	-2.66	-0.03	0.03
	(-0.92,-0.77)	(-1.44,-0.97)	(-1.3,-1.04)	(-2.79,-2.51)	(-0.05,-0.01)	(0,0.06)
2001-2011	-1.18	-1.04	0.52	2.26	0.74	-0.01
	(-1.24,-1.06)	(-1.18,-0.85)	(0.44,0.6)	(2.18,2.34)	(0.72,0.76)	(-0.03,0.01)
2012-2021	0.85	1.24	-0.34	1.75	0.46	-0.22
	(0.79,0.93)	(1.03,1.5)	(-0.43,-0.24)	(1.6,1.86)	(0.45,0.48)	(-0.26,-0.18)
ASDR(95%CI)						
1990-2021	-2.61	-4.1	-3.77	-2.5	-0.31	-1.4
	(-2.7,-2.55)	(-4.36,-3.87)	(-3.92,-3.65)	(-2.73,-2.32)	(-0.32,-0.29)	(-1.51,-1.25)
1990-2000	-3.91	-10.64	-3.81	-8.47	-0.29	0.83
	(-4.12,-3.73)	(-11.22,-10.09)	(-4.02,-3.56)	(-8.87,-8.03)	(-0.33,-0.25)	(0.44,1.31)
2001-2011	-2.62	-1.99	-4.97	1.35	0.92	-3.14
	(-2.77,-2.46)	(-2.52,-1.45)	(-5.28,-4.75)	(1.07,1.73)	(0.88,0.95)	(-3.36,-2.8)
2012-2021	-0.98	1.04	-2.85	-0.67	-1.64	-1.98
	(-1.27,-0.8)	(0.19,1.8)	(-3.37,-2.43)	(-1.45,-0.04)	(-1.69,-1.6)	(-2.22,-1.63)
ASDALR(95%CI)						
1990-2021	-2.9	-3.89	-4.27	-2.27	-0.26	-1.58
	(-3,-2.83)	(-4.12,-3.71)	(-4.35,-4.19)	(-2.45,-2.14)	(-0.27,-0.25)	(-1.74,-1.45)
1990-2000	-4.23	-9.87	-4.8	-8.67	-0.12	0.62
	(-4.45,-4.04)	(-10.32,-9.45)	(-4.94,-4.66)	(-8.97,-8.32)	(-0.16,-0.09)	(0.25,1.04)
2001-2011	-3.09	-2.21	-5.26	1.88	0.78	-3.02
	(-3.23,-2.95)	(-2.6,-1.84)	(-5.51,-5.16)	(1.62,2.2)	(0.74,0.81)	(-3.24,-2.85)
2012-2021	-1.15	1.17	-2.8	-0.34	-1.52	-2.29
	(-1.46,-0.91)	(0.41,1.85)	(-3.07,-2.46)	(-0.9,0.2)	(-1.56,-1.48)	(-2.63,-2.05)

AAPC Average annual percent change, ASPR Age-standardized prevalence rate, ASDR Age-standardized death rate, ASDALR Age-standardized disability-adjusted life years rate, CI confidence interval, HHD Hypertensive heart disease.

Table S2

AAPC of ASPR, ASDR and ASDALR for HHD in East Asia for female from 1990 to 2021

	China	Japan	South korea	China-Taiwan	North korea	Mongolia
ASPR(95%CI)						
1990-2021	-0.36	-0.52	0.12	0.47	0.54	0.03
	(-0.39,-0.33)	(-0.58,-0.45)	(0.09,0.16)	(0.43,0.5)	(0.53,0.55)	(0.01,0.04)
1990-2000	-0.95	-1.25	-0.81	-2.06	0.09	-0.37
	(-1,-0.87)	(-1.43,-1.04)	(-0.92,-0.66)	(-2.15,-1.96)	(0.07,0.1)	(-0.41,-0.34)
2001-2011	-1.35	-1.11	1.07	1.84	1.04	0.49
	(-1.42,-1.25)	(-1.22,-0.96)	(0.99,1.14)	(1.76,1.92)	(1.02,1.06)	(0.46,0.52)
2012-2021	1.47	1.02	-0.32	1.42	0.51	-0.15
	(1.36,1.56)	(0.87,1.21)	(-0.39,-0.23)	(1.32,1.52)	(0.48,0.53)	(-0.2,-0.1)
ASDR(95%CI)						
1990-2021	-2.96	-4.61	-3.47	-2.93	-0.22	-1.37
	(-3.04,-2.89)	(-4.79,-4.42)	(-3.59,-3.36)	(-3.12,-2.75)	(-0.23,-0.2)	(-1.5,-1.22)
1990-2000	-4.21	-11.04	-3.35	-7.89	-0.39	0.57
	(-4.36,-4.01)	(-11.53,-10.54)	(-3.53,-3.15)	(-8.33,-7.39)	(-0.43,-0.34)	(0.2,1.05)
2001-2011	-3.17	-1.7	-4.4	0.03	1.22	-2.28
	(-3.26,-2.99)	(-2.11,-1.2)	(-4.65,-4.23)	(-0.25,0.4)	(1.19,1.26)	(-2.4,-2.15)
2012-2021	-1.32	-0.61	-2.82	-1.15	-1.56	-2.28
	(-1.59,-1.16)	(-0.96,-0.24)	(-3.24,-2.46)	(-1.77,-0.72)	(-1.61,-1.52)	(-2.45,-2.15)
ASDALR(95%CI)						
1990-2021	-3.24	-4.34	-3.89	-3.07	-0.17	-1.76
	(-3.3,-3.19)	(-4.55,-4.15)	(-4,-3.81)	(-3.24,-2.94)	(-0.18,-0.16)	(-1.91,-1.63)
1990-2000	-4.54	-10.46	-4.15	-8.33	-0.26	0.3
	(-4.66,-4.37)	(-10.92,-10.02)	(-4.29,-3.98)	(-8.6,-8.02)	(-0.29,-0.22)	(-0.06,0.7)
2001-2011	-3.64	-2.43	-4.86	0.26	1.13	-2.72
	(-3.72,-3.5)	(-2.86,-1.99)	(-5.11,-4.74)	(0.01,0.54)	(1.1,1.16)	(-2.83,-2.62)
2012-2021	-1.34	0.52	-2.86	-1.41	-1.46	-2.72
	(-1.51,-1.23)	(-0.13,1.14)	(-3.17,-2.48)	(-1.98,-0.91)	(-1.5,-1.42)	(-3.01,-2.57)

AAPC Average annual percent change, ASPR Age-standardized prevalence rate, ASDR Age-standardized death rate, ASDALR Age-standardized disability-adjusted life years rate, CI confidence interval, HHD Hypertensive heart disease.

Table S3

AAPC of ASPR, ASDR and ASDALR for HHD in East Asia for male from 1990 to 2021

	China	Japan	South korea	China-Taiwan	North korea	Mongolia
ASPR(95%CI)						
1990-2021	-0.49	-0.24	-0.56	0.61	0.09	-0.29
	(-0.53,-0.47)	(-0.35,-0.1)	(-0.66,-0.47)	(0.56,0.66)	(0.07,0.11)	(-0.33,-0.26)
1990-2000	-0.85	-1.33	-1.82	-3.15	-0.19	0.66
	(-0.91,-0.78)	(-1.67,-0.97)	(-2.02,-1.53)	(-3.28,-2.99)	(-0.25,-0.14)	(0.57,0.75)
2001-2011	-0.97	-0.86	-0.55	2.73	0.11	-0.75
	(-1.02,-0.89)	(-1.06,-0.59)	(-0.71,-0.38)	(2.65,2.81)	(0.08,0.16)	(-0.81,-0.69)
2012-2021	0.4	1.71	0.39	2.07	0.43	-0.34
	(0.29,0.48)	(1.45,2.05)	(0.07,0.55)	(1.93,2.19)	(0.34,0.48)	(-0.44,-0.24)
ASDR(95%CI)						
1990-2021	-2.2	-3.96	-4.78	-1.85	-0.6	-1.51
	(-2.32,-2.11)	(-4.24,-3.74)	(-4.91,-4.66)	(-2.08,-1.67)	(-0.63,-0.58)	(-1.61,-1.39)
1990-2000	-3.23	-10.55	-5.6	-9.09	-0.07	0.58
	(-3.49,-3)	(-11.06,-10.06)	(-5.85,-5.3)	(-9.53,-8.6)	(-0.1,-0.03)	(0.33,0.92)
2001-2011	-2.22	-2.26	-6.56	3.27	0.07	-3.86
	(-2.42,-2.04)	(-2.69,-1.82)	(-6.82,-6.4)	(2.77,3.64)	(0.02,0.1)	(-4.16,-3.64)
2012-2021	-1.02	1.91	-2.38	0.46	-1.81	-1.15
	(-1.43,-0.73)	(0.97,2.72)	(-2.74,-1.97)	(-0.37,0.95)	(-1.92,-1.76)	(-1.47,-0.83)
ASDALR(95%CI)						
1990-2021	-2.5	-3.44	-5.13	-1.57	-0.5	-1.52
	(-2.6,-2.42)	(-3.66,-3.27)	(-5.23,-5.03)	(-1.78,-1.4)	(-0.52,-0.49)	(-1.6,-1.43)
1990-2000	-3.71	-9.32	-6.64	-8.85	0.1	0.73
	(-3.94,-3.51)	(-9.73,-8.93)	(-6.84,-6.42)	(-9.27,-8.42)	(0.07,0.15)	(0.52,1.02)
2001-2011	-2.6	-2.27	-6.45	3.78	0	-3.44
	(-2.75,-2.46)	(-2.61,-1.94)	(-6.61,-6.32)	(3.29,4.12)	(-0.06,0.04)	(-3.62,-3.27)
2012-2021	-0.98	2.16	-2.33	0.5	-1.64	-1.9
	(-1.31,-0.75)	(1.4,2.71)	(-2.62,-2.02)	(-0.21,1)	(-1.7,-1.59)	(-2.07,-1.7)

AAPC Average annual percent change, ASPR Age-standardized prevalence rate, ASDR Age-standardized death rate, ASDALR Age-standardized disability-adjusted life years rate, CI confidence interval, HHD Hypertensive heart disease.

Table S4

Temporal Trends in ASDALR for Attributable Risk Factors for HHD and PAF for Risk Factors for HHD in East Asia from 1990 to 2021

		ASR			ASP		
		1990	2021	1990-2021 AAPC	1990	2021	1990-2021 AAPC
China	All risk factors	715.81 (488.37,842.83)	292.52 (206.59,374.92)	-2.9 (-3,-2.83)	1 (0.99,1)	1 (0.99,1.01)	0 (0,0)
	Metabolic risks	715.81 (488.37,842.83)	292.52 (206.59,374.92)	-2.9 (-3,-2.83)	1 (0.99,1)	1 (0.99,1.01)	0 (0,0)
	High systolic blood pressure	715.81 (488.37,842.83)	292.52 (206.59,374.92)	-2.9 (-3,-2.83)	1 (0.99,1)	1 (0.99,1.01)	0 (0,0)
	High body-mass index	182.56 (104.75,260.36)	120.03 (71.9,180.13)	-1.41 (-1.48,-1.36)	0.25 (0.18,0.35)	0.41 (0.28,0.55)	1.55 (1.54,1.56)
	Dietary risks	583.29 (396.78,699.75)	186.13 (122.92,257.18)	-3.65 (-3.77,-3.56)	0.81 (0.73,0.88)	0.64 (0.49,0.77)	-0.79 (-0.8,-0.79)
	Diet low in fruits	370.15 (248.33,452.65)	107.77 (71.4,147.49)	-3.92 (-4.02,-3.84)	0.52 (0.43,0.59)	0.37 (0.29,0.44)	-1.09 (-1.1,-1.09)
	Diet low in vegetables	300.4 (195.67,384.89)	18.96 (7.59,34.92)	-8.56 (-8.68,-8.48)	0.42 (0.33,0.51)	0.06 (0.03,0.11)	-5.88 (-5.94,-5.85)
	Diet high in sodium	347.46 (151.98,532.42)	120.34 (47.53,205.5)	-3.38 (-3.45,-3.32)	0.48 (0.23,0.7)	0.41 (0.17,0.63)	-0.53 (-0.54,-0.52)
	Behavioral risks	589.41 (400.78,703.92)	192.79 (128.62,263.45)	-3.56 (-3.67,-3.47)	0.82 (0.74,0.89)	0.66 (0.52,0.79)	-0.71 (-0.72,-0.71)
	High alcohol use	44.47 (23.33,70.15)	21.6 (11.54,33.23)	-2.34 (-2.41,-2.26)	0.06 (0.04,0.09)	0.07 (0.05,0.11)	0.57 (0.54,0.6)
	Environmental /occupational risks	259.05 (67.59,427.96)	100.72 (26.39,172.54)	-3.04 (-3.11,-2.97)	0.36 (0.1,0.55)	0.34 (0.09,0.54)	-0.18 (-0.2,-0.15)
	High temperature	2.58 (-4.94,12.48)	1.41 (-2.23,6.02)	-1.26 (-2.18,-0.18)	0 (-0.01,0.02)	0 (-0.01,0.02)	1.56 (0.62,2.71)
	Low temperature	67.04 (45.22,80.09)	25.35 (17.21,33.46)	-3.31 (-3.55,-3.11)	0.09 (0.09,0.1)	0.09 (0.08,0.09)	-0.37 (-0.53,-0.19)
	Non-optimal temperature	69.36 (46.3,85.32)	26.64 (17.43,35.71)	-3.29 (-3.52,-3.14)	0.1 (0.08,0.11)	0.09 (0.08,0.1)	-0.26 (-0.39,-0.13)
	Other environmental risks	210.1 (0.01,388.42)	81.52 (0,157.64)	-3.04 (-3.11,-2.96)	0.29 (0,0.51)	0.28 (0,0.49)	-0.17 (-0.18,-0.16)
	Lead exposure	210.1 (0.01,388.42)	81.52 (0,157.64)	-3.04 (-3.11,-2.96)	0.29 (0,0.51)	0.28 (0,0.49)	-0.17 (-0.18,-0.16)
Japan	All risk factors	137.7 (122.78,146.02)	40.94 (35.45,44.66)	-3.89 (-4.12,-3.71)	1 (1,1)	1 (1,1)	0 (0,0)
	Metabolic risks	137.7 (122.78,146.02)	40.94 (35.45,44.66)	-3.89 (-4.12,-3.71)	1 (1,1)	1 (1,1)	0 (0,0)
	High systolic blood pressure	137.7 (122.78,146.02)	40.94 (35.45,44.66)	-3.89 (-4.12,-3.71)	1 (1,1)	1 (1,1)	0 (0,0)
	High body-mass index	41.05 (24.36,59.93)	15.53 (10.15,21.43)	-3.14 (-3.33,-2.95)	0.3 (0.19,0.43)	0.38 (0.26,0.51)	0.79 (0.78,0.81)
	Dietary risks	83.64 (67.69,100.46)	25.48 (19.92,31.29)	-3.83 (-4,-3.69)	0.61 (0.5,0.72)	0.62 (0.5,0.74)	0.08 (0.07,0.09)
	Diet low in fruits	45.15 (36.85,52.63)	16.87 (13.27,19.69)	-3.2 (-3.38,-3.04)	0.33 (0.28,0.38)	0.41 (0.34,0.48)	0.73 (0.71,0.74)
	Diet low in	25.57	7.27	-4.05	0.19	0.18	-0.14

	vegetables	(18.15,33.77)	(4.49,10.84)	(-4.24,-3.91)	(0.13,0.24)	(0.11,0.26)	(-0.18,-0.11)
	Diet high in sodium	43.56 (12.93,73.86)	10.98 (1.26,22.12)	-4.29 (-4.51,-4.07)	0.32 (0.09,0.53)	0.27 (0.03,0.53)	-0.54 (-0.55,-0.53)
	Behavioral risks	89.19 (73.39,105.04)	27.14 (21.76,32.72)	-3.83 (-4.01,-3.69)	0.65 (0.55,0.74)	0.66 (0.55,0.77)	0.07 (0.06,0.08)
	High alcohol use	15.12 (9.02,22.31)	4.53 (2.66,6.83)	-3.87 (-4.05,-3.71)	0.11 (0.06,0.16)	0.11 (0.07,0.16)	0.06 (0.03,0.08)
	Environmental/occupational risks	25.51 (13.98,37.35)	7.62 (3.69,11.61)	-3.89 (-4.07,-3.71)	0.19 (0.1,0.26)	0.19 (0.09,0.28)	0.01 (-0.06,0.1)
	High temperature	0.37 (-0.75,1.71)	0.07 (-0.18,0.36)	NA	0 (-0.01,0.01)	0 (0,0.01)	NA
	Low temperature	13.82 (12.03,15.68)	3.7 (3.11,4.23)	-3.97 (-4.49,-3.53)	0.1 (0.09,0.11)	0.09 (0.08,0.1)	-0.37 (-0.51,-0.23)
	Non-optimal temperature	14.16 (12.22,15.59)	3.76 (3.2,4.22)	-4.15 (-4.42,-3.91)	0.1 (0.09,0.11)	0.09 (0.08,0.1)	-0.35 (-0.49,-0.21)
	Other environmental risks	12.66 (0,25.3)	4.25 (0,8.49)	-3.54 (-3.7,-3.39)	0.09 (0,0.18)	0.1 (0,0.2)	0.38 (0.36,0.39)
	Lead exposure	12.66 (0,25.3)	4.25 (0,8.49)	-3.54 (-3.7,-3.39)	0.09 (0,0.18)	0.1 (0,0.2)	0.38 (0.36,0.39)
South Korea	All risk factors	284.06 (191.92,333.03)	75.33 (58.36,121.06)	-4.27 (-4.36,-4.19)	1 (1,1)	1 (1,1)	NA
	Metabolic risks	284.06 (191.92,333.03)	75.33 (58.36,121.06)	-4.27 (-4.36,-4.19)	1 (1,1)	1 (1,1)	NA
	High systolic blood pressure	284.06 (191.92,333.03)	75.33 (58.36,121.06)	-4.27 (-4.36,-4.19)	1 (1,1)	1 (1,1)	NA
	High body-mass index	79.27 (44.68,115.41)	27.47 (12.46,46.46)	-3.44 (-3.51,-3.39)	0.28 (0.18,0.4)	0.36 (0.18,0.57)	0.86 (0.85,0.86)
	Dietary risks	196.54 (126.93,247.37)	60.52 (43.81,94.99)	-3.78 (-3.88,-3.69)	0.69 (0.56,0.8)	0.8 (0.69,0.9)	0.48 (0.46,0.49)
	Diet low in fruits	121.39 (83.3,150.58)	37.72 (26.61,57.75)	-3.75 (-3.83,-3.68)	0.43 (0.36,0.49)	0.5 (0.4,0.6)	0.51 (0.47,0.53)
	Diet low in vegetables	48.55 (29.76,73.07)	27.35 (17.8,42.97)	-1.95 (-2.06,-1.83)	0.17 (0.11,0.25)	0.36 (0.25,0.49)	2.46 (2.41,2.51)
	Diet high in sodium	108.18 (27.86,186.16)	35.98 (9.8,63.82)	-3.54 (-3.64,-3.45)	0.38 (0.1,0.61)	0.48 (0.14,0.74)	0.75 (0.72,0.77)
	Behavioral risks	204.62 (132.96,254.31)	61.73 (45.04,97.1)	-3.84 (-3.95,-3.75)	0.72 (0.6,0.82)	0.82 (0.71,0.91)	0.41 (0.4,0.42)
	High alcohol use	27.79 (15.98,40.28)	6.05 (3.83,10.21)	-4.9 (-4.97,-4.83)	0.1 (0.06,0.13)	0.08 (0.05,0.11)	-0.62 (-0.64,-0.6)
	Environmental/occupational risks	79.17 (26.62,132.21)	23.83 (6.22,41.57)	-3.93 (-4.02,-3.84)	0.28 (0.1,0.43)	0.32 (0.09,0.52)	0.41 (0.34,0.46)
	High temperature	0.12 (-3.99,3.94)	0.15 (-0.91,1.2)	NA	0 (-0.01,0.01)	0 (-0.01,0.02)	NA
	Low temperature	27.52 (17.89,34.01)	6.54 (4.65,10.76)	-4.56 (-4.88,-4.33)	0.1 (0.08,0.11)	0.09 (0.07,0.1)	-0.42 (-0.58,-0.27)
	Non-optimal temperature	27.62 (16.91,35.61)	6.67 (4.29,10.89)	-4.54 (-4.77,-4.34)	0.1 (0.07,0.12)	0.09 (0.06,0.1)	-0.37 (-0.51,-0.23)
	Other environmental risks	57.09 (0,113.74)	18.86 (0,36.86)	-3.59 (-3.66,-3.53)	0.2 (0,0.38)	0.25 (0,0.47)	0.71 (0.68,0.73)
	Lead exposure	57.09 (0,113.74)	18.86 (0,36.86)	-3.59 (-3.66,-3.53)	0.2 (0,0.38)	0.25 (0,0.47)	0.71 (0.68,0.73)
China-Taiwan	All risk factors	404.88 (380.59,424.54)	198 (177.4,214.68)	-2.28 (-2.45,-2.14)	1 (1,1)	1 (1,1)	NA

	Metabolic risks	404.88 (380.59,424.54)	198 (177.4,214.68)	-2.28 (-2.45,-2.14)	1 (1,1)	1 (1,1)	NA
	High systolic blood pressure	404.88 (380.59,424.54)	198 (177.4,214.68)	-2.28 (-2.45,-2.14)	1 (1,1)	1 (1,1)	NA
	High body-mass index	136.32 (98.16,180.79)	99.27 (70.22,127.78)	-1.01 (-1.14,-0.86)	0.34 (0.24,0.44)	0.5 (0.36,0.64)	1.3 (1.28,1.33)
	Dietary risks	280.85 (235.79,325.87)	123.55 (96.55,152.36)	-2.61 (-2.77,-2.48)	0.69 (0.58,0.8)	0.62 (0.5,0.76)	-0.35 (-0.36,-0.34)
	Diet low in fruits	139.94 (114.73,163.85)	61.32 (46.57,76.94)	-2.62 (-2.81,-2.46)	0.35 (0.29,0.4)	0.31 (0.24,0.38)	-0.35 (-0.36,-0.33)
	Diet low in vegetables	141.34 (111.94,170.84)	53.14 (35.28,73.68)	-3.07 (-3.24,-2.91)	0.35 (0.28,0.42)	0.27 (0.18,0.36)	-0.87 (-0.9,-0.85)
	Diet high in sodium	123.8 (19.02,227.63)	56.97 (7.18,114.5)	-2.46 (-2.59,-2.32)	0.31 (0.05,0.56)	0.29 (0.04,0.58)	-0.2 (-0.22,-0.17)
	Behavioral risks	289.09 (246.14,331.43)	128.7 (103.01,156.43)	-2.58 (-2.73,-2.45)	0.71 (0.61,0.82)	0.65 (0.53,0.78)	-0.31 (-0.32,-0.3)
	High alcohol use	29.97 (19.36,41.64)	13.3 (8.02,19.35)	-2.59 (-2.79,-2.41)	0.07 (0.05,0.1)	0.07 (0.04,0.1)	-0.32 (-0.4,-0.23)
	Environmental/occupational risks	101.49 (18.97,174.03)	51.97 (8.32,90.69)	-2.14 (-2.29,-1.98)	0.25 (0.05,0.43)	0.26 (0.04,0.46)	0.14 (0.09,0.18)
	High temperature	-0.15 (-4.45,4.98)	0.51 (-2.78,4.41)	NA	0 (-0.01,0.01)	0 (-0.01,0.02)	NA
	Low temperature	19.27 (17.25,21.24)	8.29 (7.13,9.46)	-2.65 (-3.15,-2.26)	0.05 (0.04,0.05)	0.04 (0.04,0.05)	-0.46 (-0.73,-0.19)
	Non-optimal temperature	19.12 (13.68,25.43)	8.77 (4.97,13.36)	-2.47 (-2.96,-2.11)	0.05 (0.03,0.06)	0.04 (0.03,0.07)	-0.41 (-0.78,0.02)
	Other environmental risks	86.44 (0,163.1)	45.21 (0,86.81)	-2.06 (-2.2,-1.91)	0.21 (0,0.4)	0.23 (0,0.43)	0.22 (0.2,0.23)
	Lead exposure	86.44 (0,163.1)	45.21 (0,86.81)	-2.06 (-2.2,-1.91)	0.21 (0,0.4)	0.23 (0,0.43)	0.22 (0.2,0.23)
North Korea	All risk factors	504.08 (318.31,712.77)	465.31 (336.91,621.61)	-0.26 (-0.27,-0.24)	1 (1,1)	1 (1,1)	NA
	Metabolic risks	504.08 (318.31,712.77)	465.31 (336.91,621.61)	-0.26 (-0.27,-0.24)	1 (1,1)	1 (1,1)	NA
	High systolic blood pressure	504.08 (318.31,712.77)	465.31 (336.91,621.61)	-0.26 (-0.27,-0.24)	1 (1,1)	1 (1,1)	NA
	High body-mass index	119.56 (65.15,194.38)	177.24 (109.72,267.15)	1.28 (1.27,1.29)	0.24 (0.15,0.35)	0.38 (0.25,0.53)	1.55 (1.54,1.55)
	Dietary risks	392.47 (242.63,562.82)	365.03 (258.44,506.88)	-0.24 (-0.25,-0.23)	0.78 (0.67,0.88)	0.78 (0.67,0.87)	0.03 (0.02,0.03)
	Diet low in fruits	230.59 (135.68,332.1)	216.8 (149.75,310.94)	-0.19 (-0.2,-0.17)	0.46 (0.37,0.54)	0.47 (0.38,0.55)	0.06 (0.06,0.07)
	Diet low in vegetables	149.63 (85.65,235.76)	174.86 (109.58,265.2)	0.52 (0.5,0.56)	0.3 (0.21,0.4)	0.38 (0.28,0.48)	0.76 (0.75,0.78)
	Diet high in sodium	238.56 (90.65,417.58)	193.13 (57.08,348.17)	-0.7 (-0.71,-0.68)	0.48 (0.2,0.71)	0.42 (0.13,0.67)	-0.42 (-0.43,-0.42)
	Behavioral risks	397.2 (246.33,567.53)	369 (262.89,512.67)	-0.25 (-0.26,-0.23)	0.79 (0.68,0.88)	0.79 (0.69,0.88)	0.02 (0.02,0.02)
	High alcohol use	25.1 (12.22,42.32)	21.05 (9.78,39.43)	-0.57 (-0.59,-0.54)	0.05 (0.03,0.08)	0.05 (0.02,0.07)	-0.32 (-0.34,-0.31)
	Environmental/occupational risks	153.07 (39.24,277.22)	150.15 (38.5,264.66)	-0.06 (-0.1,-0.01)	0.3 (0.09,0.49)	0.32 (0.09,0.52)	0.18 (0.14,0.21)
	High temperature	0.22 (-4,3.82)	0.86 (-4.24,5.6)	NA	0 (-0.01,0.01)	0 (-0.01,0.01)	NA

	Low temperature	44.8 (28.56,66.07)	39.31 (26.64,54.59)	-0.53 (-0.71,-0.39)	0.09 (0.08,0.1)	0.08 (0.07,0.09)	-0.21 (-0.34,-0.09)
	Non-optimal temperature	44.98 (27.14,67.88)	40.09 (25.65,58.87)	-0.49 (-0.67,-0.34)	0.09 (0.07,0.1)	0.09 (0.06,0.1)	-0.18 (-0.31,-0.06)
	Other environmental risks	118.67 (0,242.66)	120.42 (0,236.22)	0.04 (0.03,0.05)	0.24 (0,0.44)	0.26 (0,0.48)	0.31 (0.31,0.32)
	Lead exposure	118.67 (0,242.66)	120.42 (0,236.22)	0.04 (0.03,0.05)	0.24 (0,0.44)	0.26 (0,0.48)	0.31 (0.31,0.32)
Mongolia	All risk factors	273.64 (173.11,401.15)	167.9 (114.66,237.44)	-1.58 (-1.74,-1.45)	1 (1,1)	1 (1,1)	NA
	Metabolic risks	273.64 (173.11,401.15)	167.9 (114.66,237.44)	-1.58 (-1.74,-1.45)	1 (1,1)	1 (1,1)	NA
	High systolic blood pressure	273.64 (173.11,401.15)	167.9 (114.66,237.44)	-1.58 (-1.74,-1.45)	1 (1,1)	1 (1,1)	NA
	High body-mass index	140.02 (84.12,206.58)	92.13 (57.69,136.41)	-1.34 (-1.47,-1.23)	0.51 (0.42,0.62)	0.55 (0.42,0.67)	0.24 (0.23,0.25)
	Dietary risks	204.5 (131.79,311.84)	114.71 (75.74,164.43)	-1.88 (-2.03,-1.76)	0.75 (0.65,0.83)	0.68 (0.59,0.77)	-0.28 (-0.29,-0.28)
	Diet low in fruits	122.4 (75.2,184.36)	71.28 (47.82,99.42)	-1.74 (-1.88,-1.61)	0.45 (0.38,0.52)	0.43 (0.36,0.5)	-0.16 (-0.16,-0.15)
	Diet low in vegetables	117.4 (74.24,170.63)	61.88 (39.65,93.31)	-2.08 (-2.23,-1.97)	0.43 (0.35,0.51)	0.37 (0.3,0.44)	-0.48 (-0.49,-0.48)
	Diet high in sodium	65.47 (9.18,149.31)	28.78 (1.94,78.18)	-2.63 (-2.76,-2.5)	0.24 (0.04,0.48)	0.17 (0.01,0.4)	-1.07 (-1.08,-1.05)
	Behavioral risks	207.12 (133.57,314.9)	119.13 (79.69,169.97)	-1.79 (-1.94,-1.67)	0.76 (0.66,0.83)	0.71 (0.63,0.79)	-0.2 (-0.21,-0.2)
	High alcohol use	11.6 (4.79,21.8)	14.82 (7.94,24.38)	0.76 (0.68,0.84)	0.04 (0.02,0.07)	0.09 (0.06,0.13)	2.45 (2.36,2.56)
	Environmental/occupational risks	66.92 (18.91,128.94)	38.21 (12.01,70.15)	-1.92 (-2.01,-1.8)	0.24 (0.07,0.4)	0.23 (0.07,0.37)	-0.32 (-0.35,-0.28)
	High temperature	0.02 (-0.08,0.11)	0.04 (-0.15,0.21)	2.47 (-2.38,7.51)	0 (0,0)	0 (0,0)	3.87 (-1.43,8.66)
	Low temperature	22.2 (7.85,42.51)	13.66 (5.61,24.45)	-1.92 (-2.13,-1.63)	0.08 (0.03,0.13)	0.08 (0.03,0.13)	-0.08 (-0.39,0.12)
	Non-optimal temperature	22.21 (7.77,42.62)	13.69 (5.52,24.62)	-1.9 (-2.11,-1.62)	0.08 (0.03,0.13)	0.08 (0.03,0.13)	-0.09 (-0.39,0.11)
	Other environmental risks	48.63 (0,108.36)	26.69 (0,57.21)	-1.97 (-2.05,-1.88)	0.18 (0,0.34)	0.16 (0,0.31)	-0.36 (-0.37,-0.36)
	Lead exposure	48.63 (0,108.36)	26.69 (0,57.21)	-1.97 (-2.05,-1.88)	0.18 (0,0.34)	0.16 (0,0.31)	-0.36 (-0.37,-0.36)

ASDALR Age-standardized disability-adjusted life years rate, PAF Population attributable fraction, HHD Hypertensive heart disease.

Table S5

Temporal Trends in ASDALR for Attributable Risk Factors for HHD and PAF for Risk Factors for HHD in East Asia female from 1990 to 2021

		ASR(per 100,000)			ASP		
		1990	2021	1990-2021 AAPC	1990	2021	1990-2021 AAPC
China	All risk factors	666.93 (440.3,807.97)	239.79 (157.45,351.62)	-3.24 (-3.29,-3.18)	1 (0.99,1.01)	1 (0.98,1.02)	0 (0,0)
	Metabolic risks	666.93 (440.3,807.97)	239.79 (157.45,351.62)	-3.24 (-3.29,-3.18)	1 (0.99,1.01)	1 (0.98,1.02)	0 (0,0)
	High systolic blood pressure	666.93 (440.3,807.97)	239.79 (157.45,351.62)	-3.24 (-3.29,-3.18)	1 (0.99,1.01)	1 (0.98,1.02)	0 (0,0)
	High body-mass index	179.42 (100.97,260.46)	105.21 (59.22,169.87)	-1.7 (-1.75,-1.65)	0.27 (0.19,0.36)	0.44 (0.3,0.58)	1.59 (1.58,1.6)
	Dietary risks	517.44 (323.07,642.91)	138.5 (83.93,220.85)	-4.16 (-4.22,-4.1)	0.78 (0.67,0.87)	0.58 (0.42,0.73)	-0.94 (-0.95,-0.94)
	Diet low in fruits	324.66 (190.92,431.21)	81.04 (50.82,125.37)	-4.4 (-4.47,-4.33)	0.49 (0.39,0.59)	0.34 (0.25,0.43)	-1.17 (-1.18,-1.16)
	Diet low in vegetables	258.85 (155.82,351.62)	13.14 (4.01,28.56)	-9.2 (-9.35,-9.13)	0.39 (0.29,0.48)	0.05 (0.02,0.11)	-6.15 (-6.24,-6.1)
	Diet high in sodium	283.75 (94.97,481)	83.94 (24.53,170.61)	-3.84 (-3.88,-3.78)	0.42 (0.16,0.66)	0.35 (0.11,0.59)	-0.62 (-0.62,-0.61)
	Behavioral risks	519.24 (324.73,645.19)	139.8 (85.37,222.37)	-4.14 (-4.2,-4.08)	0.78 (0.67,0.87)	0.58 (0.43,0.74)	-0.92 (-0.93,-0.92)
	High alcohol use	8.35 (2.68,15.41)	3.16 (1.37,5.89)	-3.11 (-3.18,-3.05)	0.01 (0,0.02)	0.01 (0.01,0.02)	0.14 (0.1,0.17)
	Environmental /occupational risks	212.91 (62.44,358.15)	72.85 (20.25,135.39)	-3.41 (-3.49,-3.33)	0.32 (0.1,0.5)	0.3 (0.09,0.49)	-0.21 (-0.24,-0.18)
	High temperature	2.5 (-4.52,11.61)	1.13 (-1.7,4.96)	-1.88 (-2.8,-0.79)	0 (-0.01,0.02)	0 (-0.01,0.02)	1.44 (0.51,2.6)
	Low temperature	62.69 (40.05,77.61)	20.63 (12.69,31.04)	-3.58 (-3.89,-3.35)	0.09 (0.09,0.1)	0.09 (0.08,0.09)	-0.36 (-0.5,-0.24)
	Non-optimal temperature	64.94 (40.24,83.43)	21.66 (13.32,32.66)	-3.53 (-3.78,-3.33)	0.1 (0.08,0.11)	0.09 (0.07,0.1)	-0.3 (-0.43,-0.18)
	Other environmental risks	164.04 (0.01,317.31)	56.3 (0,120.57)	-3.38 (-3.43,-3.31)	0.25 (0,0.45)	0.23 (0,0.44)	-0.16 (-0.16,-0.15)
	Lead exposure	164.04 (0.01,317.31)	56.3 (0,120.57)	-3.38 (-3.43,-3.31)	0.25 (0,0.45)	0.23 (0,0.44)	-0.16 (-0.16,-0.15)
Japan	All risk factors	131.15 (113.16,141.64)	32.62 (26,37.09)	-4.34 (-4.55,-4.15)	1 (1,1)	1 (1,1)	0 (0,0)
	Metabolic risks	131.15 (113.16,141.64)	32.62 (26,37.09)	-4.34 (-4.55,-4.15)	1 (1,1)	1 (1,1)	0 (0,0)
	High systolic blood pressure	131.15 (113.16,141.64)	32.62 (26,37.09)	-4.34 (-4.55,-4.15)	1 (1,1)	1 (1,1)	0 (0,0)
	High body-mass index	39.48 (21.26,59.38)	11.43 (5.78,17.81)	-3.87 (-4.07,-3.65)	0.3 (0.17,0.45)	0.35 (0.19,0.53)	0.5 (0.49,0.51)
	Dietary risks	76.4 (59.16,94.28)	19.69 (13.87,25.43)	-4.36 (-4.53,-4.14)	0.58 (0.47,0.7)	0.6 (0.47,0.74)	0.11 (0.09,0.12)
	Diet low in fruits	41.22 (32.75,49.02)	13.24 (9.46,16.96)	-3.67 (-3.91,-3.5)	0.31 (0.26,0.36)	0.41 (0.32,0.49)	0.83 (0.81,0.84)
	Diet low in	23.15	6.18	-4.13	0.18	0.19	0.24

	vegetables	(15.6,31.3)	(3.37,9.68)	(-4.32,-3.95)	(0.12,0.24)	(0.11,0.28)	(0.19,0.27)
	Diet high in sodium	37.79 (8.53,67.67)	7.8 (0.46,17.54)	-4.96 (-5.2,-4.73)	0.29 (0.07,0.51)	0.24 (0.02,0.51)	-0.61 (-0.63,-0.6)
	Behavioral risks	80.48 (63.72,97.58)	20.58 (14.93,26.09)	-4.41 (-4.57,-4.19)	0.61 (0.51,0.72)	0.63 (0.51,0.76)	0.08 (0.07,0.09)
	High alcohol use	10.09 (5.57,16.37)	2.25 (1.18,3.71)	-4.66 (-4.87,-4.44)	0.08 (0.04,0.12)	0.07 (0.04,0.11)	-0.33 (-0.35,-0.32)
	Environmental/occupational risks	23.56 (13.13,34.21)	6.19 (2.82,9.81)	-4.22 (-4.42,-3.97)	0.18 (0.1,0.25)	0.19 (0.09,0.29)	0.19 (0.1,0.28)
	High temperature	0.35 (-0.71,1.63)	0.05 (-0.14,0.27)	NA	0 (-0.01,0.01)	0 (0,0.01)	NA
	Low temperature	13.15 (10.96,15)	2.89 (2.2,3.42)	-4.85 (-5.15,-4.46)	0.1 (0.09,0.11)	0.09 (0.08,0.1)	-0.43 (-0.57,-0.29)
	Non-optimal temperature	13.47 (11.36,15.06)	2.93 (2.28,3.43)	-4.84 (-5.12,-4.5)	0.1 (0.09,0.11)	0.09 (0.08,0.1)	-0.41 (-0.56,-0.27)
	Other environmental risks	11.25 (0,22.5)	3.59 (0,7.29)	-3.71 (-3.91,-3.54)	0.09 (0,0.17)	0.11 (0,0.22)	0.78 (0.75,0.8)
	Lead exposure	11.25 (0,22.5)	3.59 (0,7.29)	-3.71 (-3.91,-3.54)	0.09 (0,0.17)	0.11 (0,0.22)	0.78 (0.75,0.8)
South Korea	All risk factors	264.43 (169.35,318.18)	79.48 (57.87,132.39)	-3.9 (-4,-3.81)	1 (1,1)	1 (1,1)	NA
	Metabolic risks	264.43 (169.35,318.18)	79.48 (57.87,132.39)	-3.9 (-4,-3.81)	1 (1,1)	1 (1,1)	NA
	High systolic blood pressure	264.43 (169.35,318.18)	79.48 (57.87,132.39)	-3.9 (-4,-3.81)	1 (1,1)	1 (1,1)	NA
	High body-mass index	71.16 (37.06,107)	29.76 (11.79,54.42)	-2.86 (-2.93,-2.8)	0.27 (0.16,0.4)	0.37 (0.17,0.6)	1.07 (1.06,1.07)
	Dietary risks	174.39 (106.5,223.91)	61.79 (41.52,95.34)	-3.38 (-3.5,-3.28)	0.66 (0.52,0.77)	0.78 (0.64,0.89)	0.52 (0.5,0.54)
	Diet low in fruits	104.79 (67.14,132.76)	37.57 (23.73,60.33)	-3.38 (-3.49,-3.3)	0.4 (0.33,0.46)	0.47 (0.35,0.59)	0.58 (0.53,0.62)
	Diet low in vegetables	42.46 (24.91,64.22)	27.75 (16.13,45.92)	-1.47 (-1.58,-1.37)	0.16 (0.1,0.23)	0.35 (0.23,0.48)	2.53 (2.47,2.58)
	Diet high in sodium	92.85 (19.56,169.87)	35.72 (8.62,65.76)	-3.15 (-3.24,-3.05)	0.35 (0.08,0.59)	0.45 (0.12,0.72)	0.82 (0.79,0.85)
	Behavioral risks	179.69 (110.72,228.32)	62.76 (42.56,96.87)	-3.42 (-3.53,-3.33)	0.68 (0.55,0.79)	0.79 (0.65,0.9)	0.48 (0.46,0.49)
	High alcohol use	15.39 (8.63,23.67)	3.91 (2.02,7.55)	-4.41 (-4.48,-4.34)	0.06 (0.04,0.09)	0.05 (0.03,0.08)	-0.53 (-0.54,-0.52)
	Environmental/occupational risks	66.6 (24.47,112.87)	23.64 (6.48,42.17)	-3.33 (-3.43,-3.24)	0.25 (0.1,0.39)	0.3 (0.09,0.49)	0.6 (0.54,0.65)
	High temperature	0.12 (-3.66,3.71)	0.15 (-0.93,1.26)	NA	0 (-0.01,0.01)	0 (-0.01,0.02)	NA
	Low temperature	25.52 (15.68,32.2)	6.83 (4.56,11.84)	-4.19 (-4.47,-3.96)	0.1 (0.08,0.11)	0.09 (0.07,0.1)	-0.46 (-0.6,-0.32)
	Non-optimal temperature	25.61 (14.79,34.06)	6.97 (4.31,11.87)	-4.12 (-4.35,-3.91)	0.1 (0.07,0.12)	0.09 (0.06,0.1)	-0.4 (-0.54,-0.26)
	Other environmental risks	45.37 (0,93.17)	18.31 (0,36.68)	-2.97 (-3.07,-2.87)	0.17 (0,0.33)	0.23 (0,0.44)	0.96 (0.92,0.99)
	Lead exposure	45.37 (0,93.17)	18.31 (0,36.68)	-2.97 (-3.07,-2.87)	0.17 (0,0.33)	0.23 (0,0.44)	0.96 (0.92,0.99)
China-Taiwan	All risk factors	388.78 (357.62,414.38)	148.01 (127.83,163.28)	-3.07 (-3.24,-2.94)	1 (1,1)	1 (1,1)	NA

	Metabolic risks	388.78 (357.62,414.38)	148.01 (127.83,163.28)	-3.07 (-3.24,-2.94)	1 (1,1)	1 (1,1)	NA
	High systolic blood pressure	388.78 (357.62,414.38)	148.01 (127.83,163.28)	-3.07 (-3.24,-2.94)	1 (1,1)	1 (1,1)	NA
	High body-mass index	132.23 (89.33,181.19)	75.3 (46.33,105.67)	-1.79 (-1.93,-1.67)	0.34 (0.24,0.46)	0.51 (0.32,0.69)	1.32 (1.3,1.34)
	Dietary risks	251.68 (199.94,304.18)	91.08 (63.66,117.53)	-3.23 (-3.39,-3.09)	0.65 (0.52,0.77)	0.62 (0.45,0.78)	-0.17 (-0.18,-0.16)
	Diet low in fruits	119.85 (94.93,143.89)	43.7 (29.87,58.81)	-3.2 (-3.4,-3.06)	0.31 (0.25,0.37)	0.3 (0.21,0.39)	-0.12 (-0.15,-0.09)
	Diet low in vegetables	118.76 (90.55,147.11)	38.11 (22.9,55.81)	-3.56 (-3.73,-3.41)	0.31 (0.24,0.38)	0.26 (0.16,0.38)	-0.6 (-0.64,-0.57)
	Diet high in sodium	110.21 (14.94,211.34)	45.24 (5.07,90.34)	-2.83 (-2.97,-2.7)	0.28 (0.04,0.53)	0.31 (0.03,0.61)	0.23 (0.22,0.25)
	Behavioral risks	253.71 (203.03,305.52)	91.62 (64.4,117.91)	-3.24 (-3.4,-3.1)	0.65 (0.53,0.77)	0.62 (0.46,0.78)	-0.18 (-0.19,-0.17)
	High alcohol use	5.91 (2.91,9.97)	1.39 (0.6,2.51)	-4.46 (-4.61,-4.31)	0.02 (0.01,0.03)	0.01 (0,0.02)	-1.53 (-1.56,-1.49)
	Environmental/occupational risks	78.59 (18.38,136)	35.03 (6.07,63.55)	-2.59 (-2.75,-2.43)	0.2 (0.05,0.35)	0.24 (0.04,0.42)	0.49 (0.43,0.55)
	High temperature	-0.15 (-4.22,4.77)	0.37 (-1.98,3.28)	NA	0 (-0.01,0.01)	0 (-0.01,0.02)	NA
	Low temperature	18.51 (16.38,20.58)	6.02 (5,6.9)	-3.63 (-4.21,-3.17)	0.05 (0.04,0.05)	0.04 (0.04,0.04)	-0.56 (-0.84,-0.29)
	Non-optimal temperature	18.36 (13.04,24.67)	6.37 (3.57,9.74)	-3.24 (-3.71,-2.85)	0.05 (0.03,0.06)	0.04 (0.02,0.07)	-0.51 (-0.88,-0.08)
	Other environmental risks	63.21 (0,124.93)	29.96 (0,59.69)	-2.37 (-2.51,-2.24)	0.16 (0,0.32)	0.2 (0,0.4)	0.71 (0.69,0.72)
	Lead exposure	63.21 (0,124.93)	29.96 (0,59.69)	-2.37 (-2.51,-2.24)	0.16 (0,0.32)	0.2 (0,0.4)	0.71 (0.69,0.72)
North Korea	All risk factors	487.08 (275.58,740.06)	463.32 (266.97,674.39)	-0.16 (-0.17,-0.15)	1 (1,1)	1 (1,1)	NA
	Metabolic risks	487.08 (275.58,740.06)	463.32 (266.97,674.39)	-0.16 (-0.17,-0.15)	1 (1,1)	1 (1,1)	NA
	High systolic blood pressure	487.08 (275.58,740.06)	463.32 (266.97,674.39)	-0.16 (-0.17,-0.15)	1 (1,1)	1 (1,1)	NA
	High body-mass index	119.17 (54.3,205.77)	191.41 (99.26,311.62)	1.54 (1.53,1.56)	0.24 (0.15,0.36)	0.41 (0.27,0.57)	1.72 (1.71,1.72)
	Dietary risks	369.12 (196.23,578.22)	358.02 (199.81,539.4)	-0.1 (-0.12,-0.09)	0.76 (0.61,0.88)	0.77 (0.65,0.88)	0.06 (0.06,0.06)
	Diet low in fruits	219.81 (113.96,344.91)	217.52 (117.69,337.42)	-0.03 (-0.05,-0.02)	0.45 (0.35,0.57)	0.47 (0.37,0.57)	0.13 (0.12,0.14)
	Diet low in vegetables	138.84 (70.52,239.56)	172.61 (92.02,276.53)	0.74 (0.72,0.76)	0.29 (0.2,0.4)	0.37 (0.26,0.49)	0.87 (0.86,0.89)
	Diet high in sodium	213.7 (60.19,412.53)	181.27 (36.89,367.57)	-0.54 (-0.56,-0.52)	0.44 (0.15,0.7)	0.39 (0.09,0.66)	-0.38 (-0.38,-0.37)
	Behavioral risks	370.37 (196.88,580.53)	358.93 (200.58,540.39)	-0.1 (-0.12,-0.09)	0.76 (0.62,0.88)	0.77 (0.65,0.88)	0.06 (0.06,0.06)
	High alcohol use	5.29 (1.57,10.64)	4.17 (1.24,8.51)	-0.78 (-0.8,-0.76)	0.01 (0,0.02)	0.01 (0,0.02)	-0.62 (-0.63,-0.61)
	Environmental/occupational risks	136.5 (37.88,279.4)	143.35 (35.27,269.01)	0.12 (0.08,0.16)	0.28 (0.09,0.45)	0.31 (0.09,0.5)	0.3 (0.26,0.33)
	High temperature	0.22 (-3.97,3.86)	0.87 (-4.35,5.89)	NA	0 (-0.01,0.01)	0 (-0.01,0.01)	NA

	Low temperature	43.3 (23.81,68.88)	39.14 (21.63,58.38)	-0.43 (-0.6,-0.3)	0.09 (0.08,0.1)	0.08 (0.07,0.09)	-0.22 (-0.35,-0.1)
	Non-optimal temperature	43.5 (22.4,71.46)	39.92 (20.72,61.14)	-0.39 (-0.58,-0.24)	0.09 (0.07,0.1)	0.09 (0.06,0.1)	-0.18 (-0.31,-0.06)
	Other environmental risks	102.11 (0,235.7)	113.17 (0,238.04)	0.32 (0.3,0.34)	0.21 (0,0.4)	0.24 (0,0.45)	0.5 (0.5,0.51)
	Lead exposure	102.11 (0,235.7)	113.17 (0,238.04)	0.32 (0.3,0.34)	0.21 (0,0.4)	0.24 (0,0.45)	0.5 (0.5,0.51)
Mongolia	All risk factors	249.88 (153.48,370.1)	147.19 (98.31,218.12)	-1.76 (-1.91,-1.63)	1 (1,1)	1 (1,1)	NA
	Metabolic risks	249.88 (153.48,370.1)	147.19 (98.31,218.12)	-1.76 (-1.91,-1.63)	1 (1,1)	1 (1,1)	NA
	High systolic blood pressure	249.88 (153.48,370.1)	147.19 (98.31,218.12)	-1.76 (-1.91,-1.63)	1 (1,1)	1 (1,1)	NA
	High body-mass index	134.43 (79.54,206.51)	83.02 (50.22,130.24)	-1.62 (-1.75,-1.5)	0.54 (0.43,0.65)	0.56 (0.42,0.7)	0.17 (0.16,0.18)
	Dietary risks	179.82 (107.77,275.59)	97.25 (62.74,144.18)	-2.01 (-2.14,-1.88)	0.72 (0.62,0.81)	0.66 (0.56,0.76)	-0.27 (-0.28,-0.27)
	Diet low in fruits	110.07 (64.85,166.01)	61.84 (39.67,93.71)	-1.92 (-2.05,-1.78)	0.44 (0.36,0.52)	0.42 (0.33,0.52)	-0.14 (-0.15,-0.13)
	Diet low in vegetables	105.21 (62.68,159.77)	53.41 (32.66,85)	-2.22 (-2.36,-2.09)	0.42 (0.34,0.51)	0.36 (0.27,0.45)	-0.48 (-0.49,-0.47)
	Diet high in sodium	44.96 (2.35,119.57)	18.86 (0.21,58.81)	-2.85 (-2.96,-2.73)	0.18 (0.01,0.42)	0.13 (0,0.36)	-1.08 (-1.12,-1.04)
	Behavioral risks	180.77 (108.2,277.33)	98.89 (64.77,146.28)	-1.97 (-2.1,-1.84)	0.72 (0.63,0.81)	0.67 (0.57,0.77)	-0.24 (-0.24,-0.23)
	High alcohol use	3.7 (0.94,7.57)	5.01 (2.41,9.52)	0.92 (0.84,1.01)	0.01 (0,0.03)	0.03 (0.02,0.06)	2.75 (2.7,2.82)
	Environmental/occupational risks	54.64 (16.42,109.2)	31.59 (9.92,60.48)	-1.84 (-2.01,-1.68)	0.22 (0.07,0.35)	0.21 (0.07,0.35)	-0.11 (-0.16,-0.06)
	High temperature	0.02 (-0.08,0.11)	0.03 (-0.13,0.18)	2.15 (-2.86,7.3)	0 (0,0)	0 (0,0)	3.86 (-1.43,8.63)
	Low temperature	20.28 (6.92,40.98)	11.91 (4.74,22.22)	-2.11 (-2.35,-1.77)	0.08 (0.03,0.13)	0.08 (0.03,0.13)	-0.1 (-0.4,0.1)
	Non-optimal temperature	20.29 (6.83,41.07)	11.94 (4.66,22.36)	-2.09 (-2.32,-1.78)	0.08 (0.03,0.13)	0.08 (0.03,0.13)	-0.11 (-0.42,0.09)
	Other environmental risks	37.36 (0,87.56)	21.38 (0,47.53)	-1.87 (-2,-1.74)	0.15 (0,0.3)	0.15 (0,0.29)	-0.11 (-0.12,-0.1)
	Lead exposure	37.36 (0,87.56)	21.38 (0,47.53)	-1.87 (-2,-1.74)	0.15 (0,0.3)	0.15 (0,0.29)	-0.11 (-0.12,-0.1)

ASDALR Age-standardized disability-adjusted life years rate, PAF Population attributable fraction, HHD Hypertensive heart disease.

Table S6

Temporal Trends in ASDALR for Attributable Risk Factors for HHD and PAF for Risk Factors for HHD in East Asia male from 1990 to 2021

		ASR			ASP		
		1990	2021	1990-2021 AAPC	1990	2021	1990-2021 AAPC
China	All risk factors	782.34 (515.32,947.65)	363.91 (236.12,480.99)	-2.49 (-2.6,-2.41)	1 (0.98,1)	1 (0.99,1.01)	0 (0,0)
	Metabolic risks	782.34 (515.32,947.65)	363.91 (236.12,480.99)	-2.49 (-2.6,-2.41)	1 (0.98,1)	1 (0.99,1.01)	0 (0,0)
	High systolic blood pressure	782.34 (515.32,947.65)	363.91 (236.12,480.99)	-2.49 (-2.6,-2.41)	1 (0.98,1)	1 (0.99,1.01)	0 (0,0)
	High body-mass index	187.15 (103.95,277.17)	138.86 (77.4,212.64)	-1.02 (-1.09,-0.97)	0.24 (0.16,0.33)	0.38 (0.26,0.52)	1.53 (1.52,1.53)
	Dietary risks	674.38 (435.35,821.94)	249.65 (152.94,351.3)	-3.18 (-3.28,-3.09)	0.86 (0.79,0.92)	0.69 (0.53,0.82)	-0.73 (-0.74,-0.73)
	Diet low in fruits	434.48 (284.07,546.67)	144.16 (86.16,203.86)	-3.53 (-3.63,-3.45)	0.55 (0.46,0.64)	0.4 (0.3,0.48)	-1.09 (-1.1,-1.09)
	Diet low in vegetables	359.06 (234.45,485.23)	28.06 (9.75,56.13)	-7.94 (-8.09,-7.86)	0.46 (0.37,0.56)	0.08 (0.03,0.15)	-5.61 (-5.68,-5.58)
	Diet high in sodium	433.2 (207.57,640.15)	167.13 (66.23,281.86)	-3.06 (-3.13,-2.99)	0.55 (0.28,0.75)	0.46 (0.2,0.68)	-0.6 (-0.6,-0.59)
	Behavioral risks	686.02 (441.92,837.05)	263.23 (164.49,362.59)	-3.14 (-3.24,-3.07)	0.88 (0.81,0.93)	0.72 (0.59,0.84)	-0.62 (-0.62,-0.61)
	High alcohol use	89.04 (47.67,140.06)	44.79 (23.93,68.04)	-2.23 (-2.3,-2.16)	0.11 (0.07,0.16)	0.12 (0.08,0.17)	0.25 (0.23,0.27)
	Environmental /occupational risks	322.5 (74.32,528.68)	139.63 (33.59,237.58)	-2.7 (-2.78,-2.63)	0.41 (0.1,0.63)	0.38 (0.09,0.59)	-0.25 (-0.26,-0.23)
	High temperature	2.69 (-5.23,13.4)	1.79 (-2.73,7.4)	-0.65 (-1.59,0.45)	0 (-0.01,0.02)	0 (-0.01,0.02)	1.67 (0.73,2.83)
	Low temperature	73.06 (46.6,90.41)	31.78 (19.68,42.38)	-2.84 (-3.04,-2.66)	0.09 (0.09,0.1)	0.09 (0.08,0.1)	-0.34 (-0.5,-0.17)
	Non-optimal temperature	75.48 (48.39,95.76)	33.41 (20.35,45.54)	-2.76 (-2.95,-2.59)	0.1 (0.08,0.11)	0.09 (0.08,0.11)	-0.23 (-0.36,-0.1)
	Other environmental risks	273.44 (0,493.6)	116.95 (0,219.81)	-2.72 (-2.8,-2.65)	0.35 (0,0.6)	0.32 (0,0.55)	-0.27 (-0.27,-0.26)
	Lead exposure	273.44 (0,493.6)	116.95 (0,219.81)	-2.72 (-2.8,-2.65)	0.35 (0,0.6)	0.32 (0,0.55)	-0.27 (-0.27,-0.26)
Japan	All risk factors	139.06 (131.78,143.97)	47.66 (44.28,50.2)	-3.44 (-3.66,-3.27)	1 (1,1)	1 (1,1)	0 (0,0)
	Metabolic risks	139.06 (131.78,143.97)	47.66 (44.28,50.2)	-3.44 (-3.66,-3.27)	1 (1,1)	1 (1,1)	0 (0,0)
	High systolic blood pressure	139.06 (131.78,143.97)	47.66 (44.28,50.2)	-3.44 (-3.66,-3.27)	1 (1,1)	1 (1,1)	0 (0,0)
	High body-mass index	39.96 (26.76,55.25)	19 (14.3,24.21)	-2.43 (-2.58,-2.26)	0.29 (0.2,0.39)	0.4 (0.31,0.5)	1.08 (1.07,1.11)
	Dietary risks	89.07 (73.86,104.42)	30.25 (23.94,36.46)	-3.48 (-3.67,-3.34)	0.64 (0.54,0.74)	0.63 (0.51,0.76)	-0.05 (-0.06,-0.04)
	Diet low in fruits	47.67 (39.89,54.79)	19.63 (16.22,22.75)	-2.89 (-3.06,-2.73)	0.34 (0.29,0.39)	0.41 (0.35,0.48)	0.58 (0.55,0.59)
	Diet low in	27.29	7.82	-4.02	0.2	0.16	-0.57

	vegetables	(19.84,35.74)	(4.86,11.55)	(-4.2,-3.88)	(0.14,0.26)	(0.1,0.24)	(-0.63,-0.54)
	Diet high in sodium	49.39 (18.1,78.79)	13.97 (1.99,26.29)	-4.05 (-4.27,-3.87)	0.36 (0.13,0.56)	0.29 (0.04,0.55)	-0.62 (-0.64,-0.61)
	Behavioral risks	96.66 (82.74,110.77)	32.76 (26.41,38.35)	-3.49 (-3.68,-3.34)	0.7 (0.6,0.78)	0.69 (0.57,0.79)	-0.04 (-0.05,-0.03)
	High alcohol use	21.81 (13.12,31.31)	7.03 (4.22,10.32)	-3.64 (-3.82,-3.48)	0.16 (0.09,0.22)	0.15 (0.09,0.21)	-0.2 (-0.21,-0.19)
	Environmental/occupational risks	26.8 (14.29,39.49)	8.71 (4.41,13.06)	-3.61 (-3.8,-3.43)	0.19 (0.1,0.28)	0.18 (0.09,0.27)	-0.18 (-0.28,-0.11)
	High temperature	0.38 (-0.75,1.73)	0.08 (-0.21,0.42)	NA	0 (-0.01,0.01)	0 (0,0.01)	NA
	Low temperature	13.99 (12.4,15.72)	4.37 (3.83,4.91)	-3.44 (-3.96,-2.98)	0.1 (0.09,0.11)	0.09 (0.08,0.1)	-0.34 (-0.48,-0.2)
	Non-optimal temperature	14.33 (12.78,15.51)	4.44 (3.98,4.89)	-3.66 (-3.93,-3.42)	0.1 (0.09,0.11)	0.09 (0.08,0.1)	-0.32 (-0.46,-0.17)
	Other environmental risks	13.9 (0,27.88)	4.71 (0,9.51)	-3.5 (-3.65,-3.35)	0.1 (0,0.2)	0.1 (0,0.2)	-0.05 (-0.07,-0.04)
	Lead exposure	13.9 (0,27.88)	4.71 (0,9.51)	-3.5 (-3.65,-3.35)	0.1 (0,0.2)	0.1 (0,0.2)	-0.05 (-0.07,-0.04)
South Korea	All risk factors	293.69 (148.49,356.92)	60.07 (47.92,90.41)	-5.13 (-5.23,-5.03)	1 (1,1)	1 (1,1)	NA
	Metabolic risks	293.69 (148.49,356.92)	60.07 (47.92,90.41)	-5.13 (-5.23,-5.03)	1 (1,1)	1 (1,1)	NA
	High systolic blood pressure	293.69 (148.49,356.92)	60.07 (47.92,90.41)	-5.13 (-5.23,-5.03)	1 (1,1)	1 (1,1)	NA
	High body-mass index	83.03 (38.89,120.73)	20.24 (11.88,31.98)	-4.59 (-4.66,-4.51)	0.28 (0.2,0.38)	0.34 (0.22,0.48)	0.56 (0.54,0.57)
	Dietary risks	220.42 (120.46,282.29)	51.11 (39.71,75.46)	-4.72 (-4.82,-4.62)	0.75 (0.64,0.85)	0.85 (0.75,0.93)	0.41 (0.4,0.42)
	Diet low in fruits	142.01 (74.59,183.6)	33.47 (24.32,49.45)	-4.67 (-4.76,-4.57)	0.48 (0.4,0.56)	0.56 (0.45,0.66)	0.47 (0.45,0.49)
	Diet low in vegetables	58.06 (28.5,91.49)	23.26 (14.61,34.81)	-2.98 (-3.1,-2.86)	0.2 (0.12,0.29)	0.39 (0.26,0.52)	2.21 (2.16,2.25)
	Diet high in sodium	129.32 (40.13,221.35)	32.57 (10.74,54.27)	-4.45 (-4.53,-4.38)	0.44 (0.14,0.68)	0.54 (0.19,0.8)	0.69 (0.68,0.7)
	Behavioral risks	232.33 (124.92,292.86)	52.52 (41.01,76.64)	-4.8 (-4.9,-4.7)	0.79 (0.69,0.87)	0.88 (0.78,0.94)	0.33 (0.32,0.33)
	High alcohol use	47.16 (23.46,69.03)	8.81 (5.5,14.7)	-5.41 (-5.5,-5.31)	0.16 (0.11,0.22)	0.15 (0.1,0.2)	-0.3 (-0.31,-0.29)
	Environmental/occupational risks	97.71 (28.34,165.03)	21.91 (4.99,36.56)	-4.82 (-4.93,-4.72)	0.33 (0.1,0.53)	0.37 (0.09,0.59)	0.3 (0.24,0.34)
	High temperature	0.12 (-4.18,4.3)	0.12 (-0.7,0.9)	NA	0 (-0.01,0.01)	0 (-0.01,0.02)	NA
	Low temperature	28.62 (14.28,36.83)	5.25 (3.84,7.89)	-5.43 (-5.69,-5.2)	0.1 (0.08,0.11)	0.09 (0.07,0.1)	-0.41 (-0.55,-0.26)
	Non-optimal temperature	28.72 (14.39,39.89)	5.35 (3.5,8.06)	-5.39 (-5.61,-5.19)	0.1 (0.07,0.12)	0.09 (0.06,0.11)	-0.35 (-0.5,-0.2)
	Other environmental risks	76.46 (0,149.01)	18.21 (0,33.1)	-4.62 (-4.7,-4.54)	0.26 (0,0.48)	0.3 (0,0.55)	0.5 (0.48,0.51)
	Lead exposure	76.46 (0,149.01)	18.21 (0,33.1)	-4.62 (-4.7,-4.54)	0.26 (0,0.48)	0.3 (0,0.55)	0.5 (0.48,0.51)
China-Taiwan	All risk factors	407.69 (386.55,432)	251.61 (226.8,276.39)	-1.58 (-1.78,-1.41)	1 (1,1)	1 (1,1)	NA

	Metabolic risks	407.69 (386.55,432)	251.61 (226.8,276.39)	-1.58 (-1.78,-1.41)	1 (1,1)	1 (1,1)	NA
	High systolic blood pressure	407.69 (386.55,432)	251.61 (226.8,276.39)	-1.58 (-1.78,-1.41)	1 (1,1)	1 (1,1)	NA
	High body-mass index	137.18 (104.75,178.89)	124.11 (93.58,157.22)	-0.34 (-0.52,-0.14)	0.34 (0.25,0.43)	0.49 (0.38,0.61)	1.25 (1.23,1.27)
	Dietary risks	302.64 (256.64,347.16)	159.16 (127.45,193.68)	-2.07 (-2.25,-1.91)	0.74 (0.63,0.84)	0.63 (0.51,0.76)	-0.53 (-0.54,-0.52)
	Diet low in fruits	157.74 (128.07,189.04)	80.44 (58.32,100.93)	-2.16 (-2.37,-1.96)	0.39 (0.32,0.46)	0.32 (0.24,0.4)	-0.61 (-0.64,-0.59)
	Diet low in vegetables	161.65 (125.97,197.51)	69.84 (45,96.3)	-2.61 (-2.82,-2.4)	0.4 (0.31,0.49)	0.28 (0.18,0.38)	-1.16 (-1.2,-1.13)
	Diet high in sodium	134.62 (18.47,243.23)	70.29 (8.38,142.95)	-2.07 (-2.23,-1.92)	0.33 (0.05,0.6)	0.28 (0.03,0.56)	-0.54 (-0.57,-0.51)
	Behavioral risks	316.39 (275.8,358.59)	169.39 (139.43,202.18)	-2.01 (-2.19,-1.85)	0.78 (0.68,0.87)	0.67 (0.56,0.79)	-0.47 (-0.48,-0.46)
	High alcohol use	52.52 (34.64,72.26)	26.65 (16.26,38.31)	-2.17 (-2.38,-1.97)	0.13 (0.08,0.17)	0.11 (0.06,0.15)	-0.61 (-0.63,-0.59)
	Environmental/occupational risks	122.82 (19.26,209.25)	71.27 (11.13,125.57)	-1.76 (-1.92,-1.58)	0.3 (0.05,0.51)	0.28 (0.04,0.48)	-0.22 (-0.26,-0.19)
	High temperature	-0.15 (-4.46,5.04)	0.66 (-3.65,5.8)	NA	0 (-0.01,0.01)	0 (-0.01,0.02)	NA
	Low temperature	19.39 (17.34,21.58)	10.72 (9.16,12.35)	-1.75 (-2.29,-1.32)	0.05 (0.04,0.05)	0.04 (0.04,0.05)	-0.38 (-0.66,-0.11)
	Non-optimal temperature	19.24 (13.69,25.7)	11.35 (6.38,17.25)	-1.56 (-2.08,-1.18)	0.05 (0.03,0.06)	0.05 (0.03,0.07)	-0.34 (-0.71,0.08)
	Other environmental risks	108.69 (0,198.32)	62.74 (0,117.27)	-1.76 (-1.92,-1.59)	0.27 (0,0.49)	0.25 (0,0.46)	-0.22 (-0.24,-0.2)
	Lead exposure	108.69 (0,198.32)	62.74 (0,117.27)	-1.76 (-1.92,-1.59)	0.27 (0,0.49)	0.25 (0,0.46)	-0.22 (-0.24,-0.2)
North Korea	All risk factors	523.97 (294.72,725.09)	448.08 (285.33,745.93)	-0.51 (-0.53,-0.49)	1 (1,1)	1 (1,1)	NA
	Metabolic risks	523.97 (294.72,725.09)	448.08 (285.33,745.93)	-0.51 (-0.53,-0.49)	1 (1,1)	1 (1,1)	NA
	High systolic blood pressure	523.97 (294.72,725.09)	448.08 (285.33,745.93)	-0.51 (-0.53,-0.49)	1 (1,1)	1 (1,1)	NA
	High body-mass index	116.26 (58.41,189.56)	142.08 (79.56,270.36)	0.66 (0.63,0.67)	0.22 (0.14,0.32)	0.32 (0.22,0.43)	1.16 (1.16,1.16)
	Dietary risks	430.21 (242.81,616.13)	363.87 (226.05,615.33)	-0.53 (-0.55,-0.52)	0.82 (0.73,0.9)	0.81 (0.71,0.9)	-0.03 (-0.04,-0.03)
	Diet low in fruits	247.77 (141.75,376.94)	208.52 (123.63,358.79)	-0.55 (-0.57,-0.54)	0.47 (0.39,0.56)	0.47 (0.37,0.56)	-0.04 (-0.05,-0.04)
	Diet low in vegetables	168.91 (92.82,264.95)	173.5 (95.81,305.73)	0.09 (0.07,0.12)	0.32 (0.23,0.42)	0.39 (0.29,0.49)	0.58 (0.57,0.6)
	Diet high in sodium	282.99 (124.33,487.26)	208.76 (72.32,423.75)	-0.98 (-1,-0.97)	0.54 (0.27,0.76)	0.47 (0.18,0.71)	-0.47 (-0.47,-0.46)
	Behavioral risks	441.08 (248.7,624.71)	372.45 (230,627.62)	-0.54 (-0.56,-0.53)	0.84 (0.76,0.91)	0.83 (0.74,0.91)	-0.04 (-0.04,-0.04)
	High alcohol use	60.72 (29.76,99.24)	46.58 (21.31,89.93)	-0.86 (-0.89,-0.84)	0.12 (0.07,0.17)	0.1 (0.06,0.15)	-0.34 (-0.35,-0.33)
	Environmental/occupational risks	183.8 (41.39,327.46)	158.7 (37.27,331.25)	-0.46 (-0.5,-0.43)	0.35 (0.09,0.56)	0.36 (0.09,0.57)	0.04 (0,0.07)
	High temperature	0.19 (-4.01,3.65)	0.82 (-4.38,5.58)	NA	0 (-0.01,0.01)	0 (-0.01,0.01)	NA

	Low temperature	46.51 (26.04,67.41)	37.83 (23.62,65.29)	-0.77 (-1.02,-0.59)	0.09 (0.08,0.1)	0.08 (0.07,0.09)	-0.21 (-0.34,-0.09)
	Non-optimal temperature	46.67 (26.67,69.09)	38.56 (22.24,69.13)	-0.72 (-1,-0.52)	0.09 (0.07,0.1)	0.09 (0.06,0.1)	-0.18 (-0.31,-0.06)
	Other environmental risks	150.53 (0,294.27)	131.43 (0,300.77)	-0.44 (-0.46,-0.42)	0.29 (0,0.52)	0.3 (0,0.53)	0.09 (0.08,0.09)
	Lead exposure	150.53 (0,294.27)	131.43 (0,300.77)	-0.44 (-0.46,-0.42)	0.29 (0,0.52)	0.3 (0,0.53)	0.09 (0.08,0.09)
Mongolia	All risk factors	307.07 (192.39,458.67)	193.6 (126.03,281.86)	-1.52 (-1.6,-1.43)	1 (1,1)	1 (1,1)	NA
	Metabolic risks	307.07 (192.39,458.67)	193.6 (126.03,281.86)	-1.52 (-1.6,-1.43)	1 (1,1)	1 (1,1)	NA
	High systolic blood pressure	307.07 (192.39,458.67)	193.6 (126.03,281.86)	-1.52 (-1.6,-1.43)	1 (1,1)	1 (1,1)	NA
	High body-mass index	146.7 (84.78,226.34)	101.71 (61.44,154.92)	-1.18 (-1.26,-1.08)	0.48 (0.39,0.58)	0.52 (0.42,0.63)	0.32 (0.3,0.34)
	Dietary risks	239.75 (148.58,365.94)	137.41 (87.73,202.78)	-1.83 (-1.92,-1.72)	0.78 (0.69,0.87)	0.71 (0.61,0.8)	-0.3 (-0.3,-0.3)
	Diet low in fruits	140.77 (83.39,220.73)	83.35 (52.26,121.17)	-1.73 (-1.81,-1.63)	0.46 (0.39,0.53)	0.43 (0.36,0.5)	-0.19 (-0.2,-0.19)
	Diet low in vegetables	135.52 (82.79,210.09)	72.83 (44.47,111.31)	-2.01 (-2.1,-1.91)	0.44 (0.36,0.52)	0.38 (0.3,0.45)	-0.51 (-0.52,-0.5)
	Diet high in sodium	93.56 (17.56,201.48)	42.69 (4.15,104.58)	-2.55 (-2.66,-2.43)	0.3 (0.07,0.55)	0.22 (0.02,0.46)	-1.01 (-1.04,-0.99)
	Behavioral risks	244.4 (151.6,374.55)	145.68 (92.65,213.07)	-1.72 (-1.8,-1.62)	0.8 (0.71,0.88)	0.75 (0.67,0.83)	-0.17 (-0.18,-0.17)
	High alcohol use	21.13 (8.39,39.61)	28.32 (14.61,45)	0.92 (0.85,1)	0.07 (0.03,0.11)	0.15 (0.09,0.21)	2.52 (2.47,2.58)
	Environmental/occupational risks	84.34 (21.95,166.96)	47.53 (14.13,85.15)	-1.93 (-2.02,-1.82)	0.28 (0.07,0.45)	0.25 (0.08,0.4)	-0.38 (-0.44,-0.34)
	High temperature	0.02 (-0.09,0.13)	0.04 (-0.17,0.25)	2.27 (-2.69,7.32)	0 (0,0)	0 (0,0)	3.89 (-1.4,8.68)
	Low temperature	24.92 (8.67,48.44)	15.85 (6.53,28.11)	-1.65 (-1.83,-1.42)	0.08 (0.03,0.13)	0.08 (0.04,0.13)	-0.07 (-0.37,0.14)
	Non-optimal temperature	24.93 (8.58,48.57)	15.88 (6.44,28.27)	-1.62 (-1.81,-1.37)	0.08 (0.03,0.13)	0.08 (0.03,0.13)	-0.07 (-0.37,0.13)
	Other environmental risks	64.63 (0,139.04)	34.46 (0,71.77)	-2.06 (-2.15,-1.95)	0.21 (0,0.39)	0.18 (0,0.34)	-0.54 (-0.56,-0.52)
	Lead exposure	64.63 (0,139.04)	34.46 (0,71.77)	-2.06 (-2.15,-1.95)	0.21 (0,0.39)	0.18 (0,0.34)	-0.54 (-0.56,-0.52)

ASDALR Age-standardized disability-adjusted life years rate, PAF Population attributable fraction, HHD Hypertensive heart disease.

Descriptive title for supplementary figures

Figure S1

Temporal trends in ASR of HHD in East Asia female from 1990 to 2021. (A) Age standardized prevalence rate. (B) Age standardized death rate. (C) Age standardized DALYs rate. ASR Age-standardized rates, HHD Hypertensive heart disease, DALYs Disability-adjusted life years

Figure S2

Temporal trends in ASR of HHD in East Asia male from 1990 to 2021. (A) Age standardized prevalence rate. (B) Age standardized death rate. (C) Age standardized DALYs rate. ASR Age-standardized rates, HHD Hypertensive heart disease, DALYs Disability-adjusted life years

Figure S3

Trends in the prevalence rates of HHD in East Asia by age group, 2012-2021

Figure S4

Trends in the mortality rates of HHD in East Asia by age group, 2012-2021

Figure S5

Trends in disability-adjusted life years for HHD in East Asia by age group, 2012-2021

Figure S6

The percentage of HHD prevalence, death, and DALYs in each age group compared to the total number. **(A)** Prevalence by HHD. **(B)** DALYs by HHD. **(C)** Death by HHD. DALYs disability-adjusted life years, HHD Hypertensive heart disease

Figure S7

East Asian HHD death cases in 2021, by age and sex. The crude death rates and their 95% uncertainty intervals are shown in the line graphs. The male and female prevalence composition by age group are displayed in pie charts.

Figure S8

East Asian HHD DALYs in 2021, by age and sex. The crude DALYs rates and their 95% uncertainty intervals are shown in the line graphs. The male and female prevalence composition by age group are displayed in pie charts.

Figure S9

DALYs attributable to age-grouped level 1 risk factors in East Asia female in 1990 and 2021

Figure S10

DALYs attributable to age-grouped level 1 risk factors in East Asia male in 1990 and 2021.

Figure S11

PAF of risk factors for DALYs due to HHD in East Asia female and male, 1990 and 2021. (A) 1990. (B) 2021.

Figure S12

PAF of risk factors for DALYs due to HHD in East Asia, 1990 and 2021. **(A)** 1990. **(B)** 2021. DALYs Disability-adjusted life years, PAF Population attributable fraction, HHD Hypertensive heart disease

Figure S13

Association Between SDI and Disease Burden:

Figure S1

Click here to access/download;Supplementary Files;Fig S1-female.png

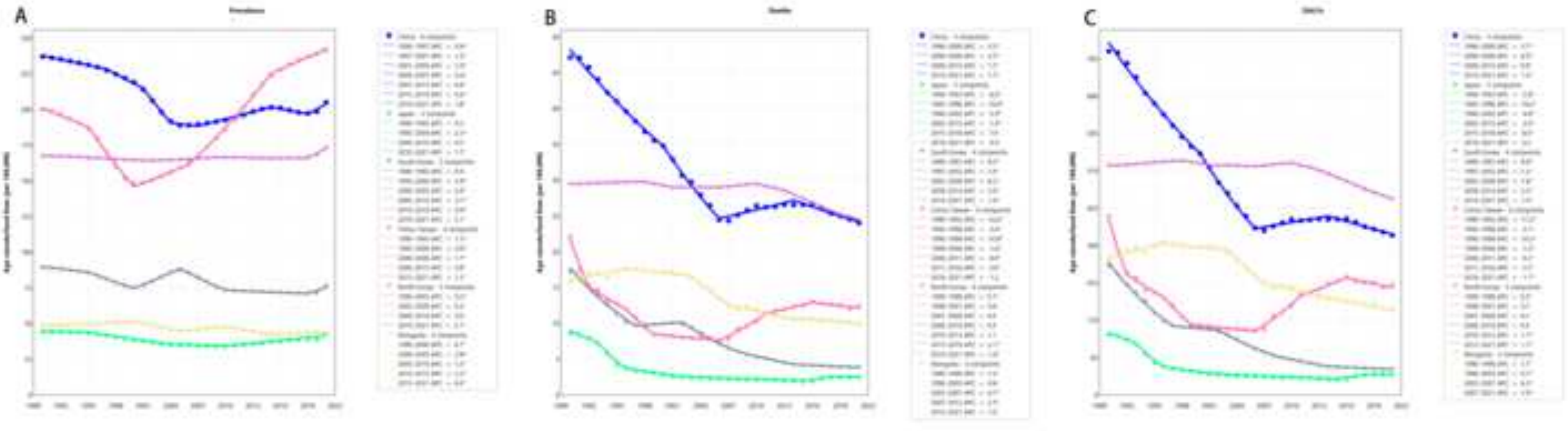

Figure S2

Click here to access/download;Supplementary Files;Fig S2- male.png

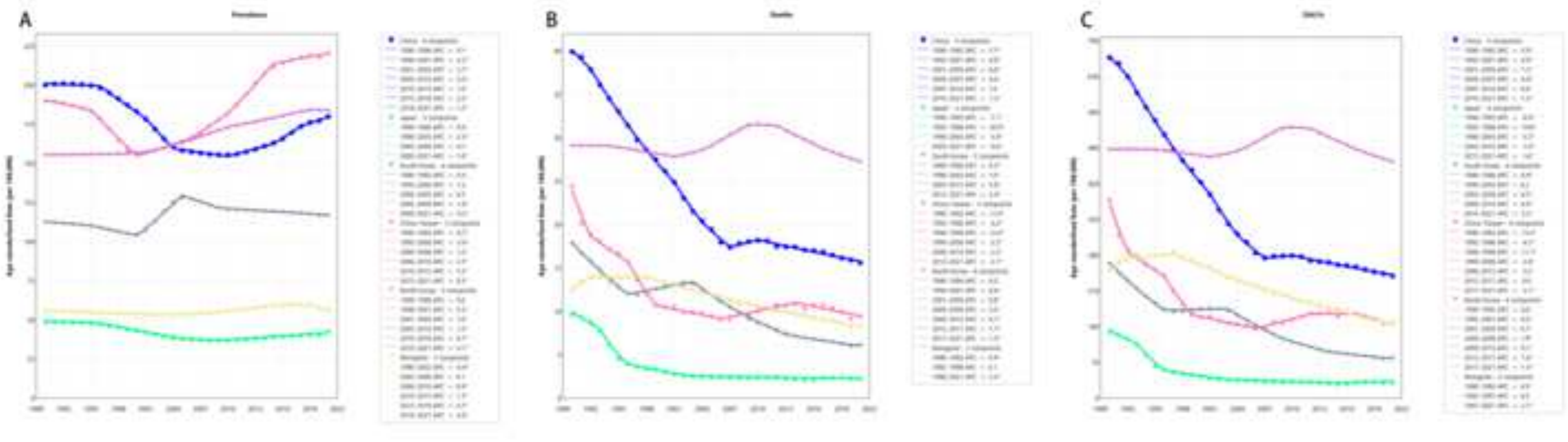

Figure S3 Click here to access/download;Supplementary Files;Figure S3.png

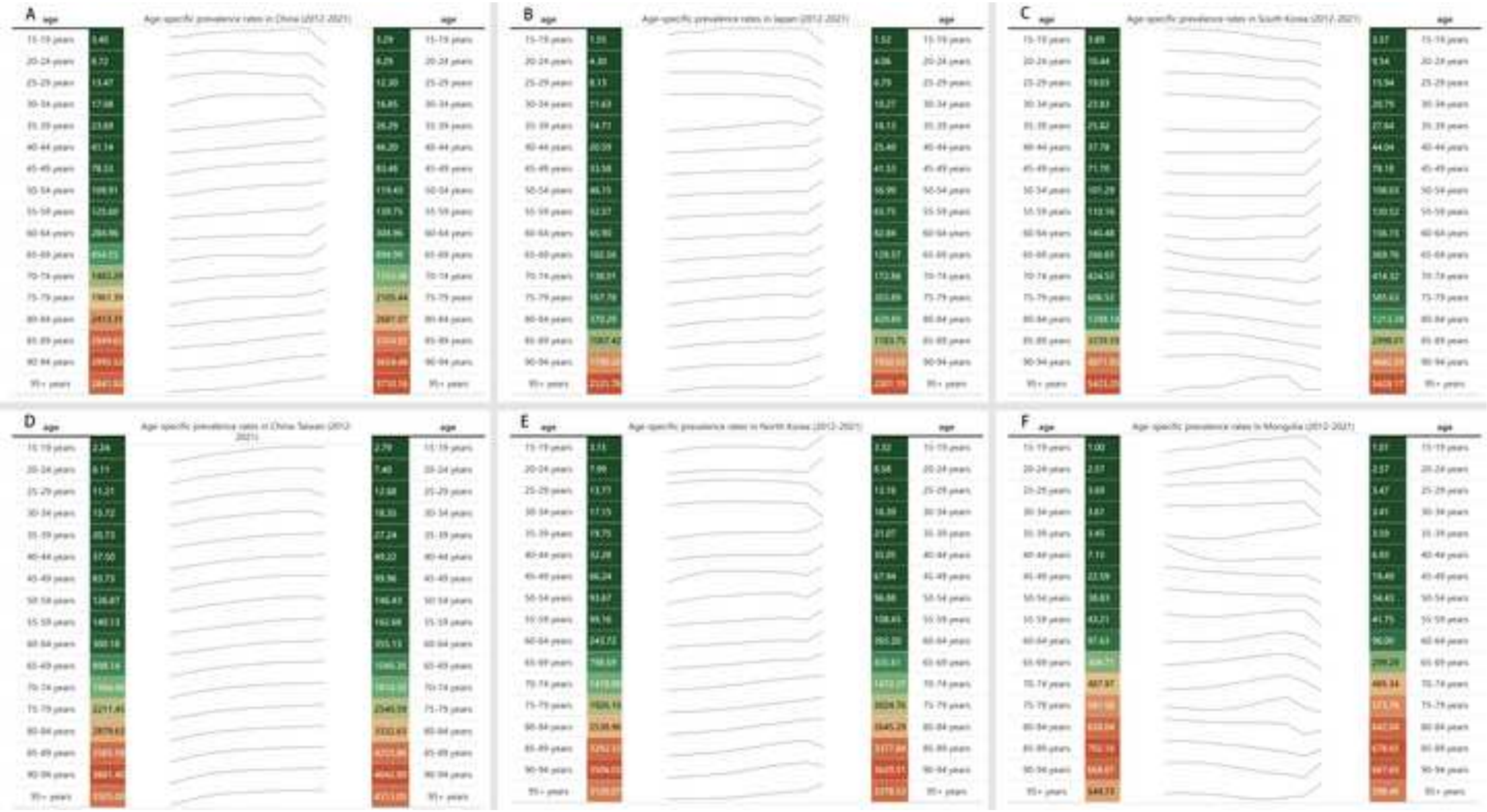

Figure S4

Click here to access/download;Supplementary Files;Figure S4.png

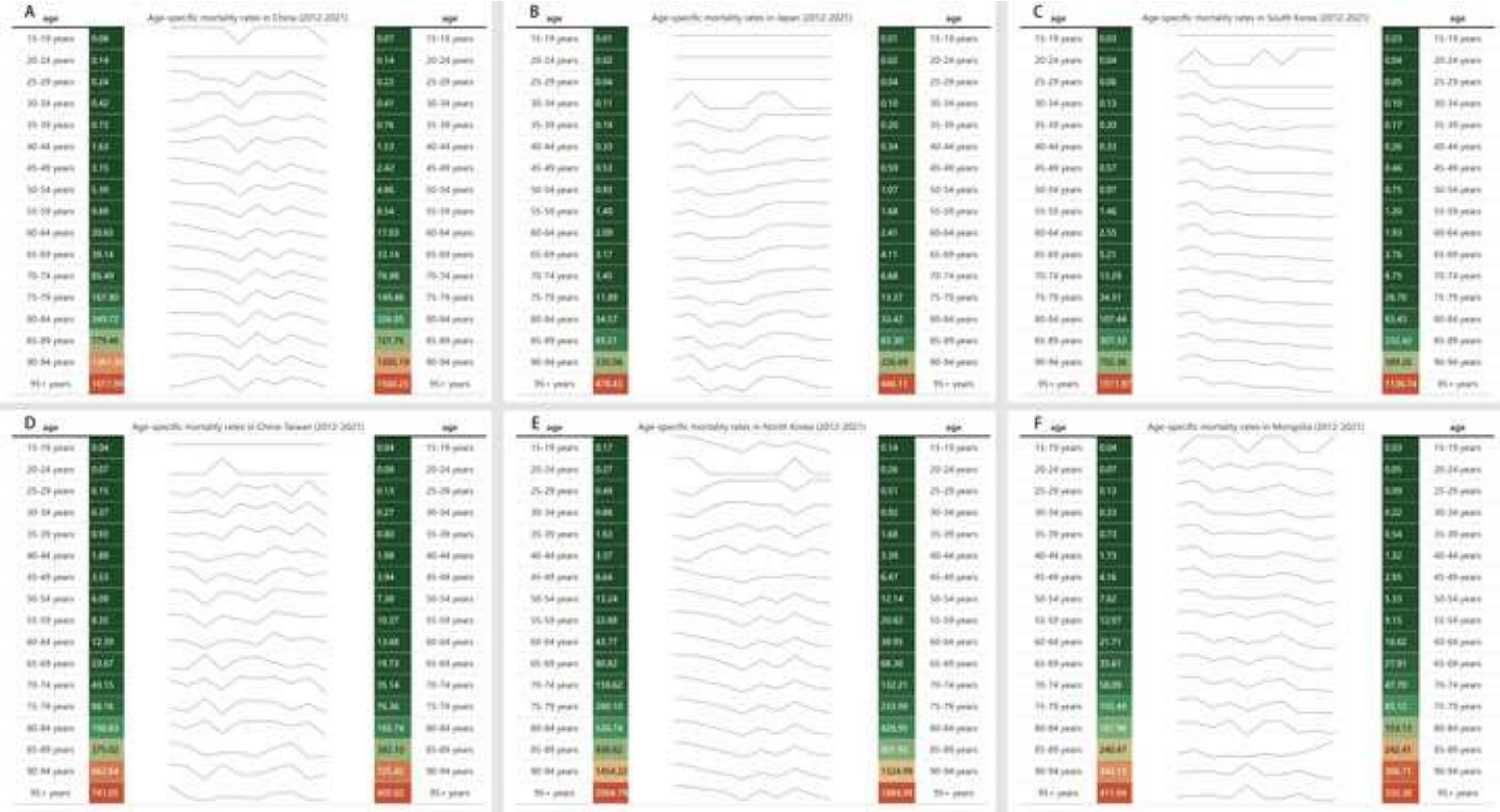

Figure S5

Click here to access/download;Supplementary Files;Figure S5.png

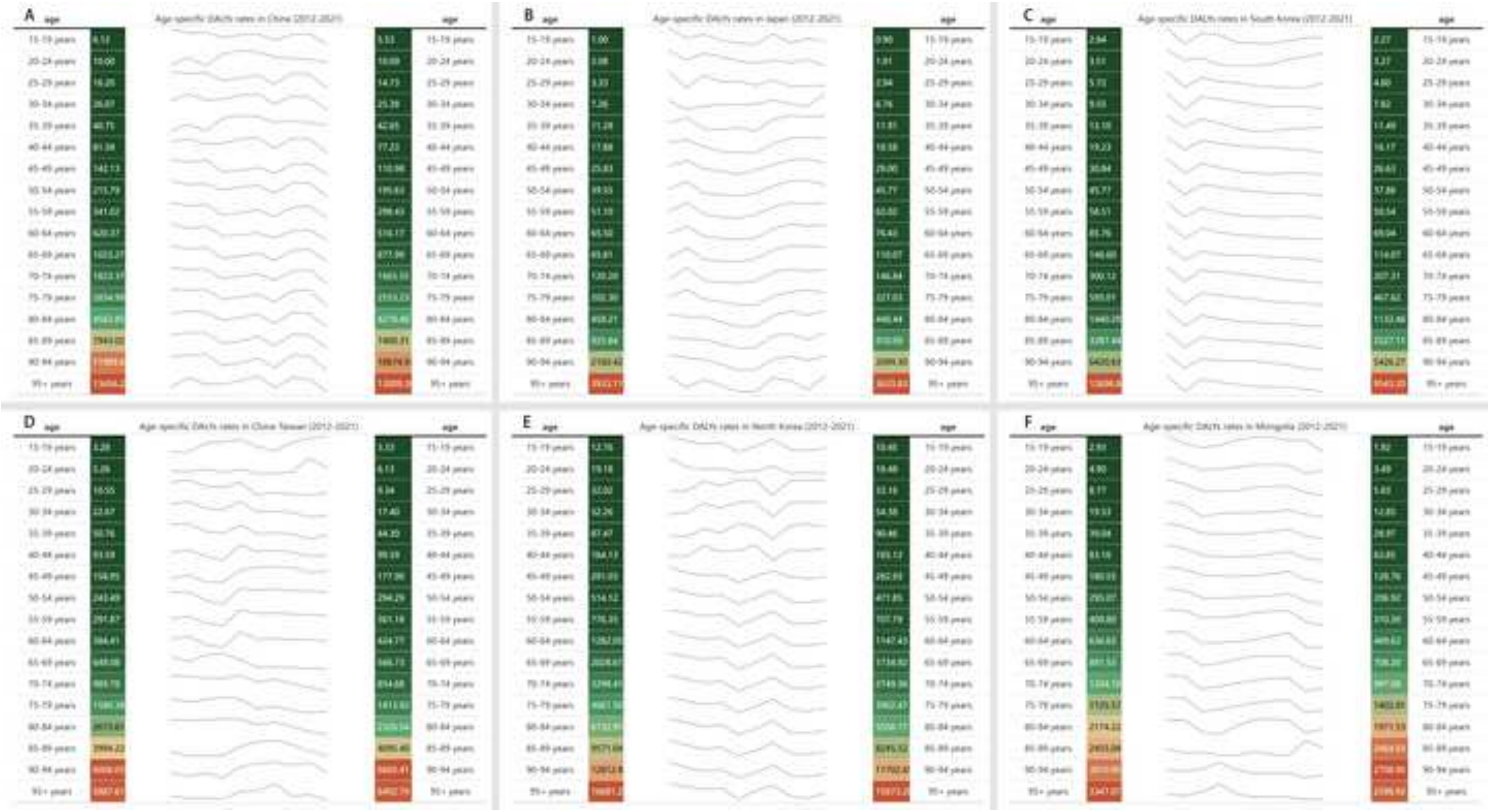

Figure S6

Click here to access/download;Supplementary Files;Fig S6_eastasia_all.png

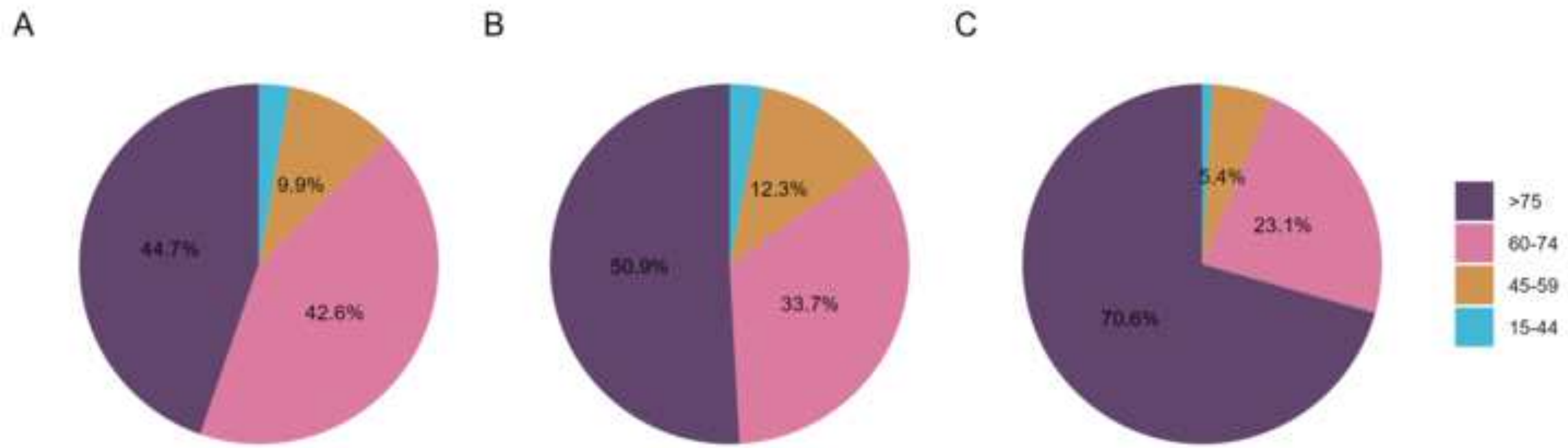

Figure S7

Click here to access/download;Supplementary Files;Fig S7-DALYs.png

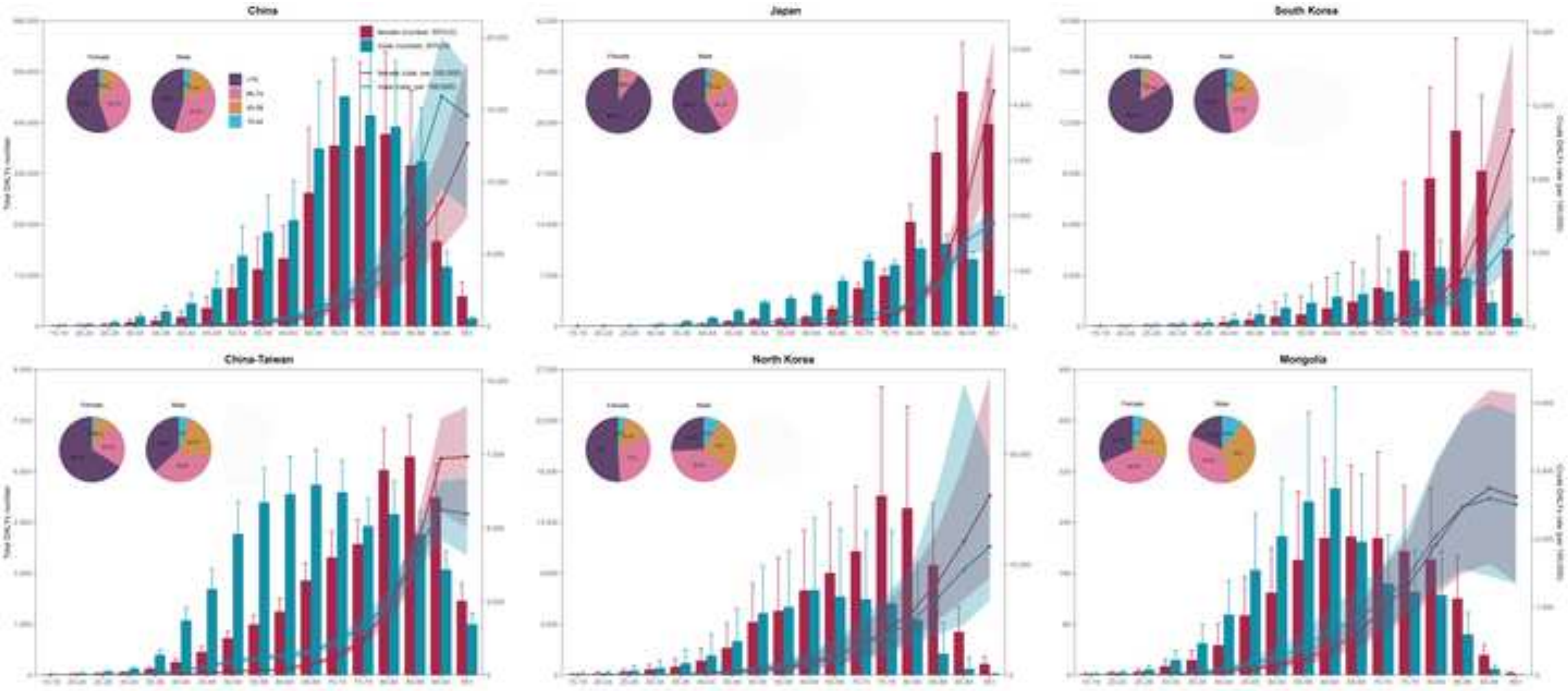

Figure S8

Click here to access/download;Supplementary Files;Fig S8-Deaths.png

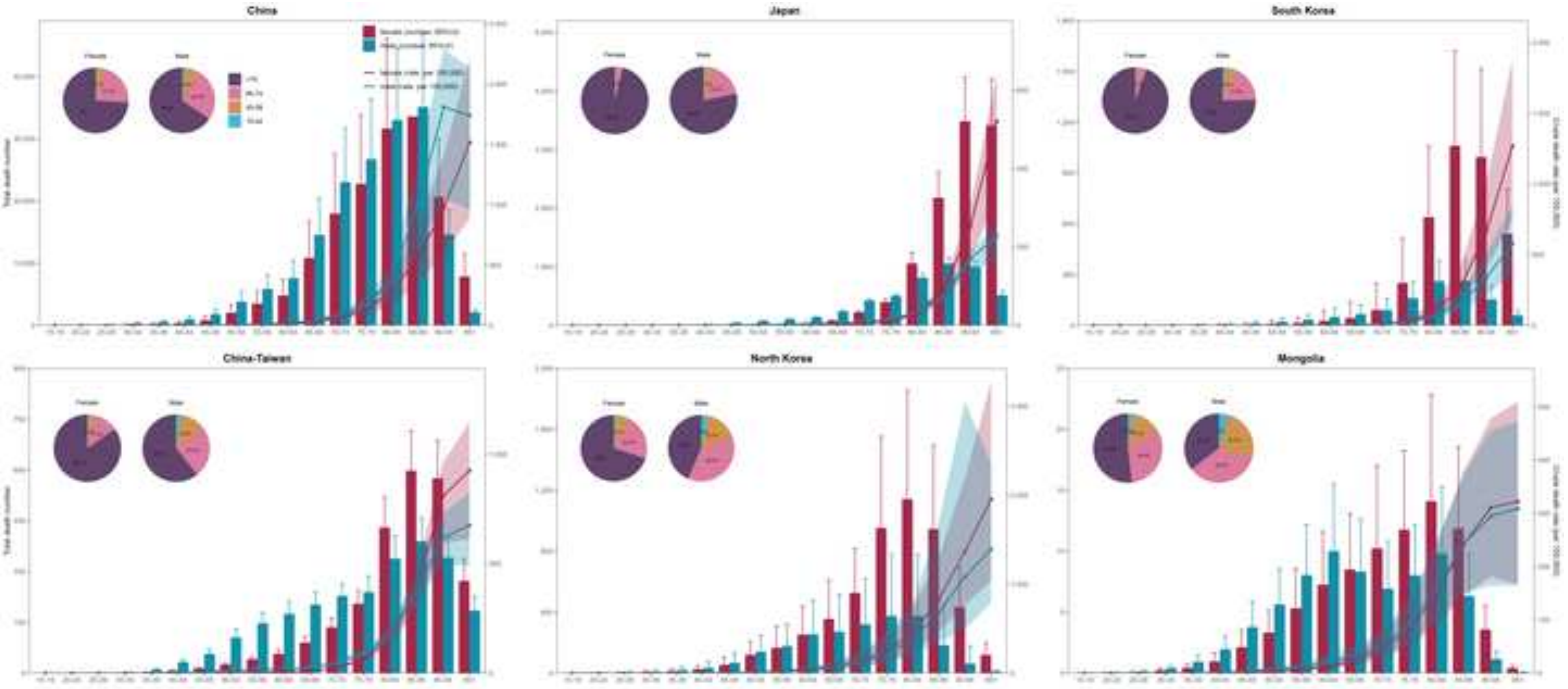

Figure S9

Click here to access/download;Supplementary Files;Fig S9-female.png

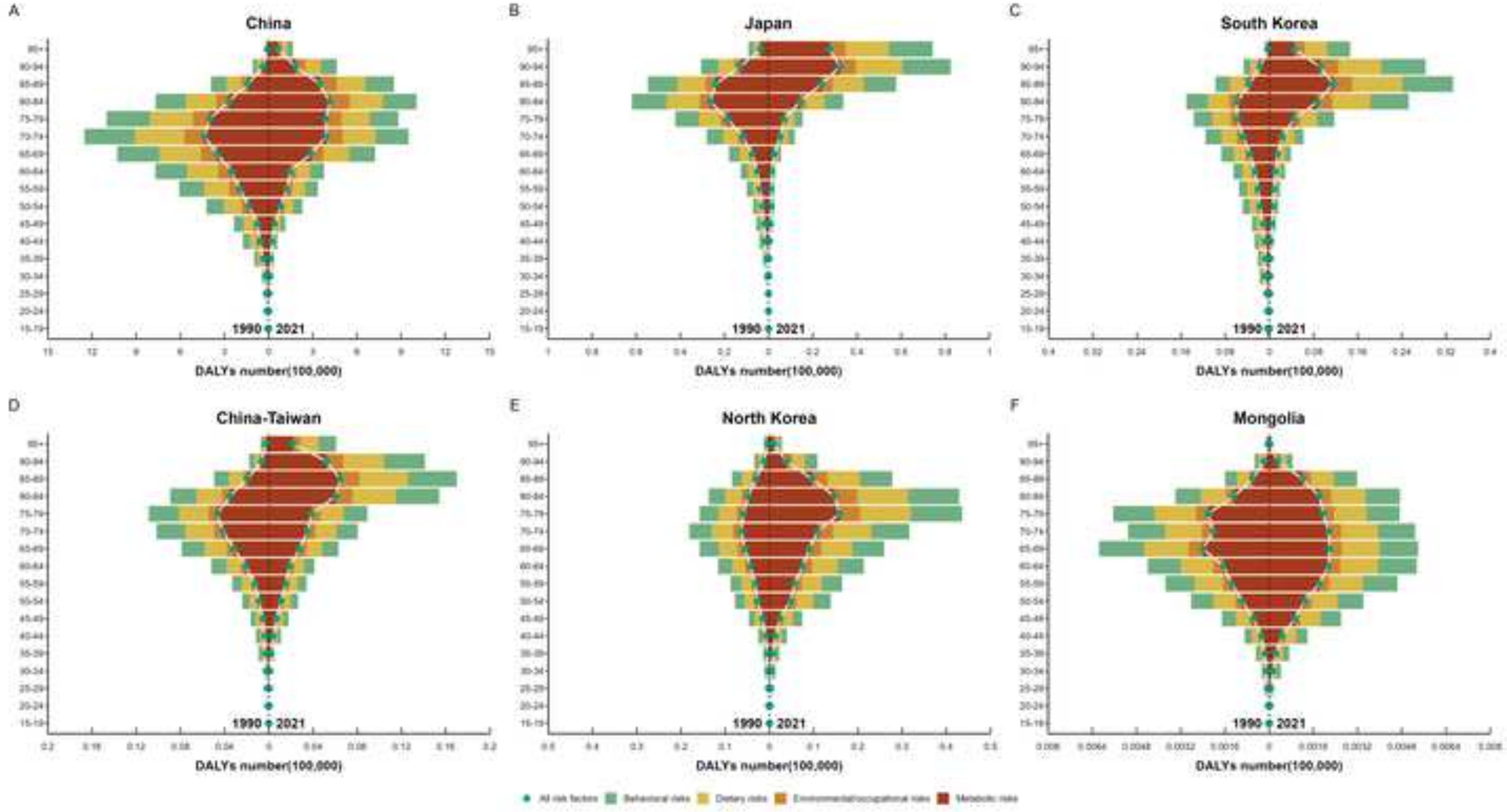

Figure S10

Click here to access/download;Supplementary Files;Fig S10-male.png

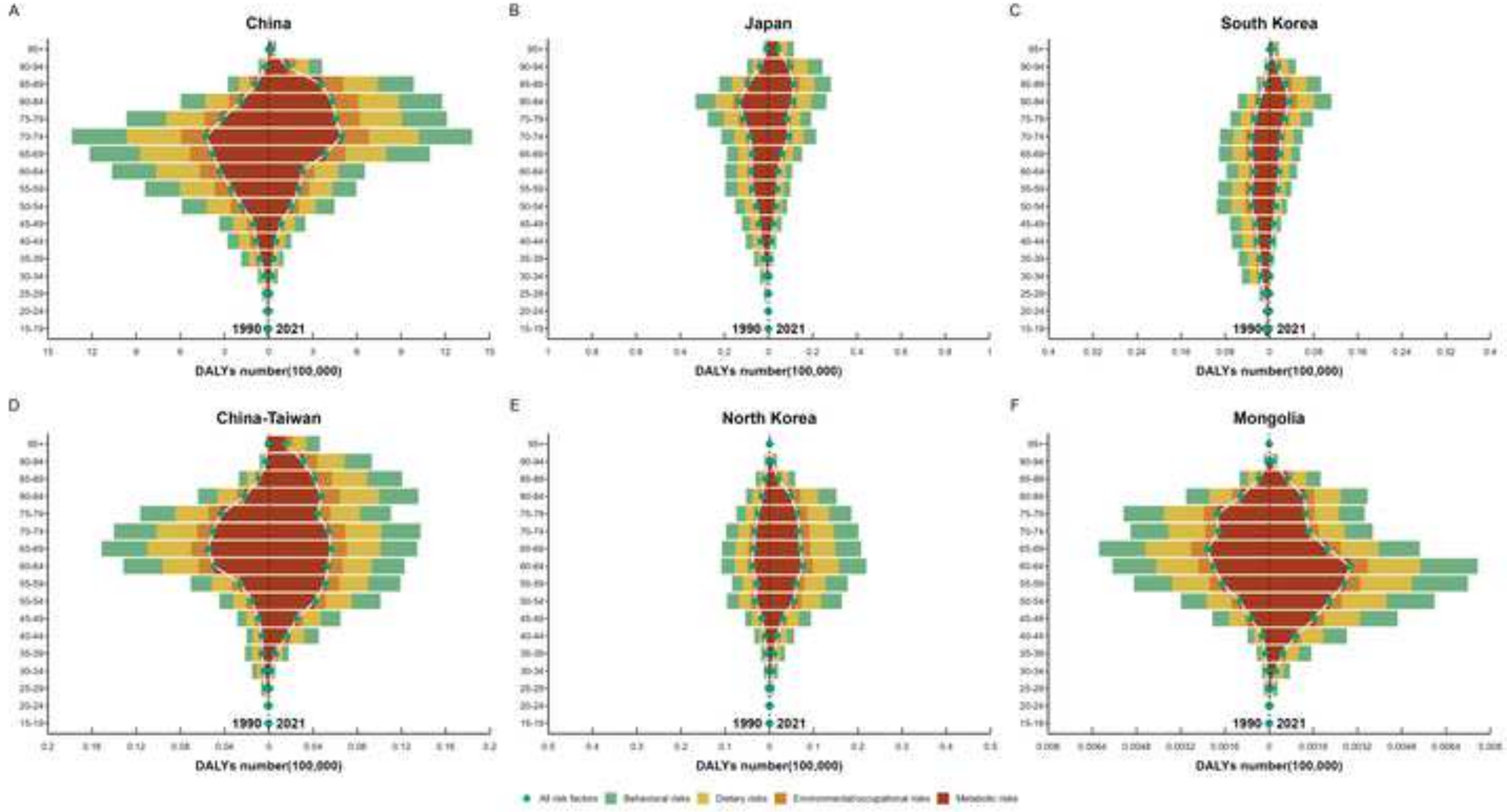

Figure S11

Click here to access/download;Supplementary Files;Fig S11-femal&male.png

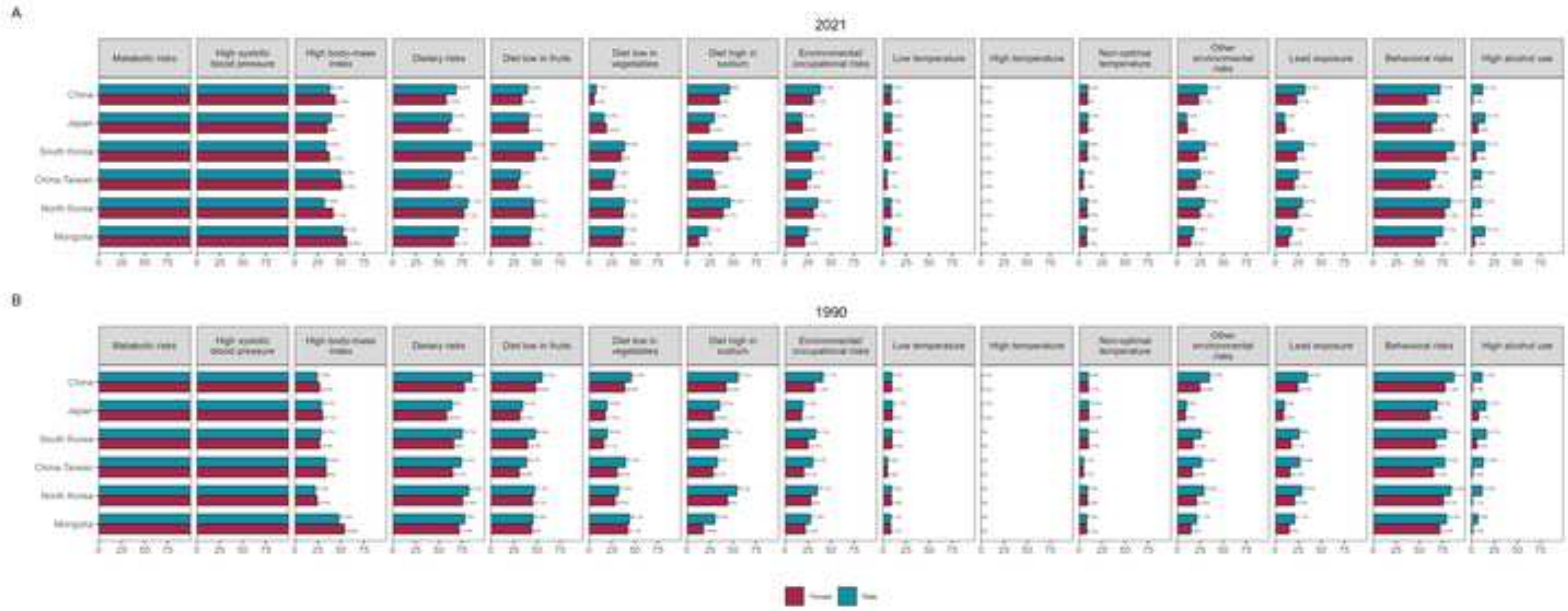

Figure S12

Click here to access/download;Supplementary Files;Fig S12-both_1990_2021.png

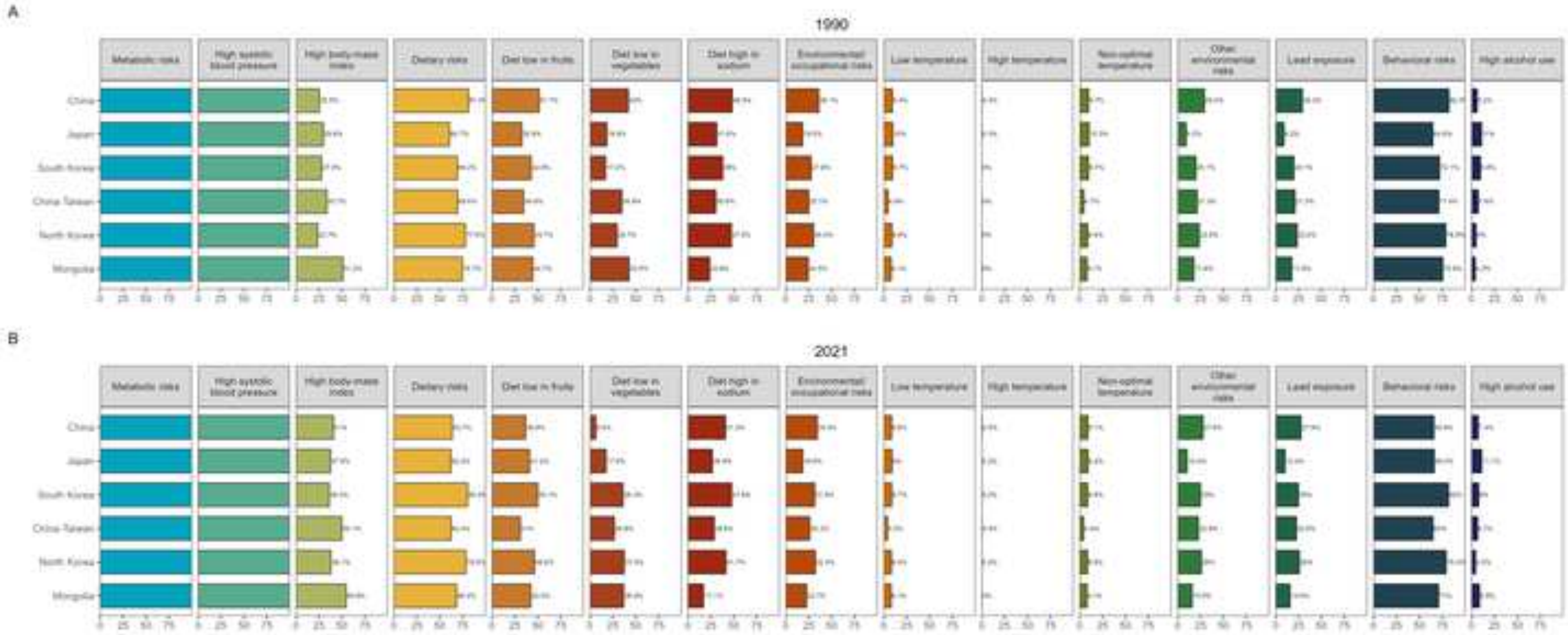

Figure S13

Click here to access/download;Supplementary Files;Fig S13_SDI.png

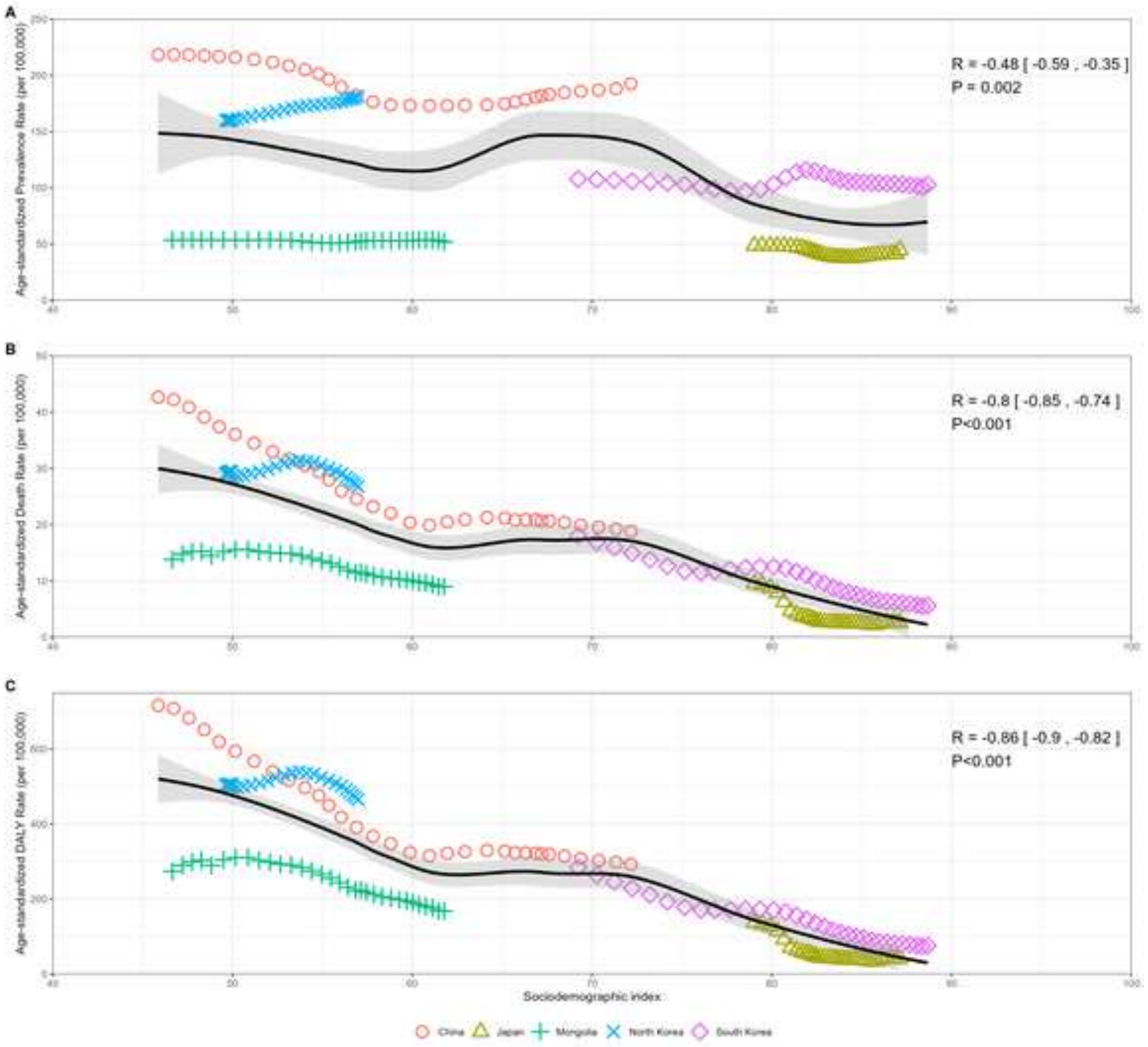

Supplement: Supplementary Files. — Table s1 to s6 and Figures s1 to s13. [file gh-20-1-1472-s1.pdf]
